# IPGCA: A Comprehensive Single Cell Atlas of 1 074 127 Porcine Intestinal Cells Revealing Cellular Dynamics, Genetic Regulation, and Cross‐Species Conservation

**DOI:** 10.1002/advs.202507882

**Published:** 2025-10-05

**Authors:** Pengfei Yu, Qinqin Xie, Xiaodian Cai, Zhanming Zhong, Ran Wei, He Han, Shuang Liu, Zhenyang Zhang, Lingyao Xu, Zitao Chen, Zhe Zhang, Qishan Wang, Yuchun Pan, Zhen Wang, Zhe Zhang

**Affiliations:** ^1^ Department of Animal Science College of Animal Sciences Zhejiang University Hangzhou 310058 China; ^2^ Key Laboratory of Livestock and Poultry Resources Evaluation and Utilization Ministry of Agriculture and Rural Affairs Hangzhou 310030 China; ^3^ State Key Laboratory of Swine and Poultry Breeding Industry Guangdong Provincial Key Lab of Agro‐Animal Genomics and Molecular Breeding College of Animal Science South China Agricultural University Guangzhou 510642 China; ^4^ Xianghu Laboratory Hangzhou 310027 China; ^5^ Hainan Institute Zhejiang University Yongyou Industrial Park Yazhou Bay Sci‐Tech City Sanya 572000 China

**Keywords:** enrichment analysis, gut, ieQTL, integrative analysis, pig, PigGTEx, scRNA‐seq

## Abstract

The porcine intestinal tract is vital for nutrient absorption, immune regulation, and various physiological processes. However, a comprehensive understanding of its cellular composition and gene regulatory landscape remains limited. Here, the Integrated Pig Gut Cell Atlas (IPGCA) is presented, a comprehensive single cell resource comprising 1 074 127 cells spanning five intestinal segments, six developmental stages, and nine breeds. IPGCA enables the discovery of previously overlooked cell types (e.g., Paneth cells) and reveals dynamic B cell remodeling during weaning. Combining with genome‐wide association studies (GWAS), complex traits are linked to potential causal cell types. Furthermore, the utility of IPGCA as a reference for the deconvolution of bulk RNA‐seq data is demonstrated, enabling the identification of cell type interaction expression quantitative trait loci (ieQTLs) and deconvolutional eQTLs (decon‐eQTL). Co‐localization analyses implicate enterocyte *BNC2* functions in loin muscle area trait and endothelial cell *BAG3* in backfat thickness. Strikingly, comparative analysis with human intestinal atlases reveals a high degree of conservation, reinforcing the pig's value as a model for human gut biology. To empower the community, scGutDB (http://alphaindex.zju.edu.cn/scgut) is developed, an interactive platform for data exploration. IPGCA provides a foundational resource for agricultural genomics, precision breeding, and translational studies of intestinal physiology and diseases.

## Graphical abstract

A high resolution integrated cell atlas of the pig intestine provides insights into the genetic mechanisms of complex traits (Created in BioRender. Yu, P. (2025) https://BioRender.com/o14c563)

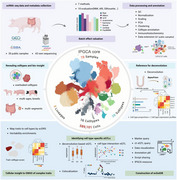

## 1 Introduction

Pigs have been regarded as invaluable meat resources for humans, and their significance has been further amplified in biomedical research as an exceptional model organism^[^ ^1^ ^]^ and an appropriate candidate for xenotransplantation^[^ ^2^ ^]^ From a biomedical perspective, pigs, like humans, are monogastric animals with comparable digestive system anatomy. The intestine, a central organ in digestion, holds paramount importance as it provides a niche for microbiota, and exerts a pivotal influence on immune modulation, metabolic functions, and the preservation of the intestinal barrier.^[^ ^3^, ^4^ ^]^ The intestinal tract's functional complexity has also rendered it integral to a spectrum of diseases and complex traits, including the gut‐brain axis, microbial interactions, and various complex economic traits in various species.^[^ ^5^ ^]^

Previous studies have made efforts to elucidate the genetic mechanisms of complex traits at the gene expression level by transcriptome sequencing (RNA‐seq).^[^ ^6^, ^7^ ^]^ For instance, integrating tissue‐level gene expression data with GWAS results enables the identification of causal tissues using heritability enrichment approaches.^[^ ^8^ ^]^ This integration also aids in interpreting associated signals within noncoding regions, as demonstrated by expression quantitative trait loci (eQTL) mapping in initiatives like GTEx^[^ ^9^ ^]^ and FarmGTEx.^[^ ^10^, ^11^, ^12^ ^]^ However, bulk RNA‐seq falls short in cellular specificity, which is a substantial constraint when dealing with cellular heterogeneity in tissues. To overcome this limitation, single cell RNA sequencing (scRNA‐seq) has emerged as a powerful tool, providing detailed insights into cellular heterogeneity and enabling higher‐resolution analysis of genetic regulation.^[^ ^13^ ^]^

In recent years, rapid expansion in both the size and quantity of scRNA‐seq datasets for the pig intestine has created an opportunity to delve into the cellular mechanisms underlying the functionality of the porcine intestinal tract. Integrated single cell atlases provide novel insights not obtained in individual studies and have led to the discovery of rare cell types and facilitated comparative analysis, revealing new biological patterns.^[^ ^14^, ^15^, ^16^, ^17^, ^18^, ^19^ ^]^ A comprehensive atlas also demonstrates its strength in associating complex traits. On one hand, we can investigate the association between cell types and complex traits by calculating enrichment scores, using tool such as scDRS.^[^ ^20^ ^]^ On the other hand, we can perform cell types specific eQTL mapping to further elucidate these genetic mechanisms.^[^ ^17^ ^]^ However, the high cost of large‐scale scRNA‐seq analysis poses a challenge to the widespread use of cell types specific eQTL mapping.^[^ ^21^ ^]^ An alternative strategy involves identifying cell type specific eQTLs from bulk tissue RNA‐seq data through computational deconvolution methods that estimate the proportions^[^ ^22^ ^]^ or gene expression levels^[^ ^23^ ^]^ of various cell types. For instance, Kim‐Hellmuth et al (2020)^[^ ^21^ ^]^ modeled an additional interaction term between genotypes and deconvolutional cell type proportions from bulk RNA‐seq data of GTEx project to identify interaction eQTL (ieQTL), revealing that *KREMEN1* influences birth weight in mice through interactions with adipocytes. In contrast, the deconvolutional gene expression can be directly considered as a phenotype for mapping cell type‐specific eQTL (decon‐ct‐eQTL), similar to what CSeQTL did.^[^ ^24^ ^]^ Both approaches require a comprehensive atlas to obtain accurately estimated cell type proportions and cell‐specific expression.^[^ ^18^ ^]^

In this study, we present a comprehensive porcine intestinal single cell atlas (Integrated pig gut cell atlas, IPGCA), and precisely re‐annotated the cell type at three levels (lineage, cell type, and sub‐cell type) by integrating existing and newly generated scRNA‐seq datasets. The IPGCA expands our understanding of pig intestinal tract, elucidating its dynamic changes associated with growth, spatial distribution, and variations across diverse genetic and pathological backgrounds. Furthermore, by integrating the IPGCA with either GWAS summary statistics or bulk RNA‐seq data, we illustrate the capacity of the IPGCA to enhance the annotation of GWAS outcomes and to act as a deconvolution reference panel, thereby enabling the precise identification of cell type‐specific genetic regulators. Ultimately, we have established an interactive online platform, scGutDB (http://alphaindex.zju.edu.cn/scgut), designed to promote the dissemination and sharing of IPGCA data resources, including the atlas markers, eQTLs, ct‐ieQTLs, and decon‐ct‐eQTLs, which help enhance the understanding of the intricate relationships between genetic variations and phenotypic traits. Additionally, the IPGCA can be readily extended using newly generated datasets through the CellTypist^[^ ^25^ ^]^ and scANVI^[^ ^26^ ^]^ pipeline, making it a valuable resource for querying, annotating, mapping complex traits, and using deconvolution references at the single cell level in pigs.

## 2 Results

### 2.1 Data Integration Establishes the IPGCA Core and Extend Landscape

**Figure 1:**
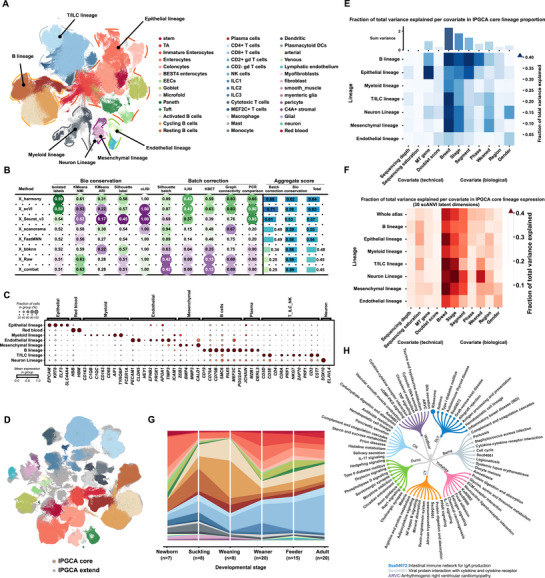
Construction of IPGCA core and extend. A) An UMAP visualization of 8 cell lineages and 38 subcell types embedding 589101 cells that consisted IPGCA core. B) Benchmarking of batch effects correction across 7 integration methods for the separately annotated atlas. C) Classical cell type markers of each cell lineage (The color showed the mean expression and the dot size showed the proportion of cells expressing gene). D) IPGCA extends integration with the IPGCA core by CellTypist and scANVI pipeline, the extended datasets are shown in grey color. E) The fraction of total inter‐sample proportion variance in the IPGCA core accounted by specific covariates. Covariates are categorized into technical (left) and biological (right). F) The fraction of total inter‐sample expression variance in the IPGCA core accounted by specific covariates. Covariates are categorized into technical (left) and biological (right). G) Distribution of IPGCA core cell types in each stage. H) The KEGG terms of breed associated DEGs. (LY: Landrace × Yorkshire; DLY: Duroc × Landrace × Yorkshire).

We constructed the comprehensive single cell atlas of the pig intestinal tracts from seven publicly available datasets (n_sample = 35) and our own data from Chinese indigenous (CN) pigs (n_sample = 28), Duroc pigs (n_sample = 10) and wild boar (n_sample = 5) using 10× Genomics scRNA‐seq platform (Table S1, Supporting Information). The datasets encompass a total of 78 porcine intestinal samples from 32 individuals across 7 different pig breeds, covering a total of 6 developmental stages ranging from newborn to adult. The sampling tissues covered 5 intestinal segments, that is, duodenum, jejunum, ileum, cecum, and colon. After cell filtration, a total of 58 9101 high‐quality cells were captured for further analysis (**Figure** **1**A), which was denoted as IPGCA core.

To remove potential batch effects across different datasets, we evaluated the bio‐conservation and batch effect correction of seven integration methods using the benchmark pipeline scib.^[^ ^27^ ^]^ As a result, Harmony^[^ ^28^ ^]^ and scVI^[^ ^26^ ^]^ have demonstrated the highest performance levels with aggregate scores of 0.64 and 0.63, respectively while scVI excels in the batch correction scores and offers the possibility for further atlas expansion (Figure 1B). Most previous studies^[^ ^18^, ^29^ ^]^ have benchmarked batch effects correction methods on human datasets, and we found that the methods exhibited a preference for specific species and tissues (**Extend** Figure 1A,B, Supporting Information). Finally, we employed the scVI^[^ ^26^ ^]^ integration technique to correct the batch effect, ensuring accurate downstream annotation.

There is an absence of consensus for the annotation of cell types derived from these datasets across various studies. To enable supervised data integration and downstream integrated analysis, we achieved a unified annotation of cell types at three levels, based on markers in original texts and established canonical markers^[^ ^27^, ^30^, ^31^, ^32^, ^33^, ^34^, ^35^, ^36^, ^37^, ^38^ ^]^ (Figure 1C; Figure S1B and Table S2, Supporting Information; Experimental Section). In conclusion, we established the IPGCA core in the identification of 8 cell lineages, 16 cell types, and 42 sub cell types for level 1–3 annotation, respectively (Figure 1A; Figure S1A, Supporting Information).

For future scalability, mapping new data to the IPGCA core enables rapid data annotation and interpretation. To demonstrate this capability, we integrated seven newly generated public datasets^[^ ^39^, ^40^, ^41^, ^42^, ^43^, ^44^, ^45^ ^]^ into the IPGCA core using CellTypist^[^ ^25^ ^]^ and scANVI^[^ ^26^ ^]^ (Figure 1D; Figure S1C, Supporting Information; Experimental Section). This process expanded the IPGCA to a total of 1074127 cells from 145 samples. The newly added datasets are referred to as IPGCA extend.

### 2.2 Biological and Technical Factors Impact Cell Type Proportions and Gene Expression

Biological and technical covariates can influence cellular proportions and transcriptional phenotypes. To better understand the impact of these covariates, we employed a linear model to assess their contributions to proportion and transcriptional variance in IPGCA core. The covariates included technical factors such as sequencing depth, sequencing saturation, fraction of mitochondrial transcripts, and doublet score, as well as biological factors such as developmental stage, anatomical spatial segment, breed, cell cycle phase, weaning status, and gender.

For cellular proportion, breed, developmental stage, and intestinal segment contributed most to the variation in proportion. Specifically, breed has a substantial impact on the variation of immune, neuronal, and mesenchymal lineages (Figure 1E). Different intestinal segments significantly influence the variation of epithelial and B cell lineages proportion, while weaning status particularly affect the immune and neuronal cells (Figure 1E). We subsequently validated these findings using the IPGCA extended dataset (Figure S1E, Supporting Information), which incorporated additional technical covariates, including sequencing platform and sequencing method. Notably, different sequencing methods (snRNA‐seq versus scRNA‐seq) affect the capture of epithelial, immune, and mesenchymal cell lineages (Figure S1E, Supporting Information). Additionally, different sequencing platforms also have some impact on certain cell lineages (Figure S1E, Supporting Information). We indeed observed significant variation in the proportions of cell types across different gastrointestinal segments, developmental stages, and breeds. For instance, the B cell lineage exhibits age‐related fluctuations, with activated B cells, cycling B cells, and plasma cells reaching their peak at the weaning stage (Figure 1G). Compared with other tissues, the ileum is distinguished by its markedly higher abundance of B lineage cells (Figure S1D, Supporting Information). Regarding breeds, a notably increased proportion of T and innate lymphoid cells (T/ILC) is observed in wild boars (Figure S1D, Supporting Information).

For transcriptional expression, developmental stage and breed are the biological variables that explain most of the variance between samples (Figure 1F; Figure S1F, Supporting Information). Additionally, breed is strongly associated with expression variation in the epithelial and T/ILC lineages, while weaning status particularly affects the variability of immune and neuronal cells, which are consistent with the conclusions drawn from proportion variance (Figure 1E; Figure S1E, Supporting Information). These associations provided a systematic overview of the biological and technical factors influencing the proportion variation and transcriptional variance of gut cell types.

Epithelial cells, which form the intestinal barrier, exhibit heterogeneity in gastrointestinal absorptive and digestive functions,^[^ ^37^ ^]^ as their cellular component and expression levels are significantly influenced by breed (Figure 1E,F; Figure S1E,F, Supporting Information). Although we confirmed that the relative proportions of epithelial subtypes are breed‐biased and display distinct segment‐specific distributions (**Extend** Figure 1C, Supporting Information), potential sampling biases across datasets preclude a definitive interpretation at the compositional level. We therefore focused our subsequent analyses on transcriptional rather than proportional differences. To further compare the differences in epithelial cells across breeds, we performed Gene Ontology (GO) and Kyoto Encyclopedia of Genes and Genomes (KEGG) enrichment analyses on their breed‐associated differentially expressed genes (DEGs, Figure 1H; Table S3, Supporting Information). “Type II diabetes mellitus (ssc04930)” were enriched among the epithelial cell‐specific expressed genes in Duroc pigs. Greater capacity for carbohydrate absorption and metabolism within CN population was illustrated by increased expression of genes in the “Starch and sucrose metabolism (ssc00500)” and “Carbohydrate digestion and absorption (ssc04973)” terms. In contrast, the Yorkshire population exhibited strengths in protein metabolism, as evidenced by upregulation of genes in the “Protein digestion and absorption (ssc04974)” and “Glycine, serine and threonine metabolism (ssc00260)” terms. Additionally, wild boars showed enhanced fatty acid metabolism, with upregulation of genes in the “Cholesterol metabolism (ssc04979)” and “Taurine and hypotaurine metabolism (ssc00430)” terms. Moreover, the terms related to the contraction and rhythm of musculus cardiacus (ssc04270, ssc05412; including natriuretic peptide and vasopressin receptor genes, e.g., *NPPB, ADRA1A*) were up‐regulated in the wild boar population. In summary, we identified differential expression of immune‐related terms among epithelial cells of various pig breeds, with distinct characteristics observed: commercial pigs primarily exhibited enhanced protein and fat absorption, while the Chinese indigenous pig breed (CN) showed greater capacity for sugar and starch metabolism. These findings aid in elucidating the mechanisms underlying the germplasm characteristics of different pig breeds, as the primary results are consistent with the known phenotypic characteristics of different pig breeds,^[^ ^46^, ^47^ ^]^ yet IPGCA can provide novel insights into the differences in digestion, absorption, and immune functions from a single cell perspective.

### 2.3 IPGCA Enables the Recovery of Previously Overlooked Cell Types Like Paneth Cells

**Figure 2:**
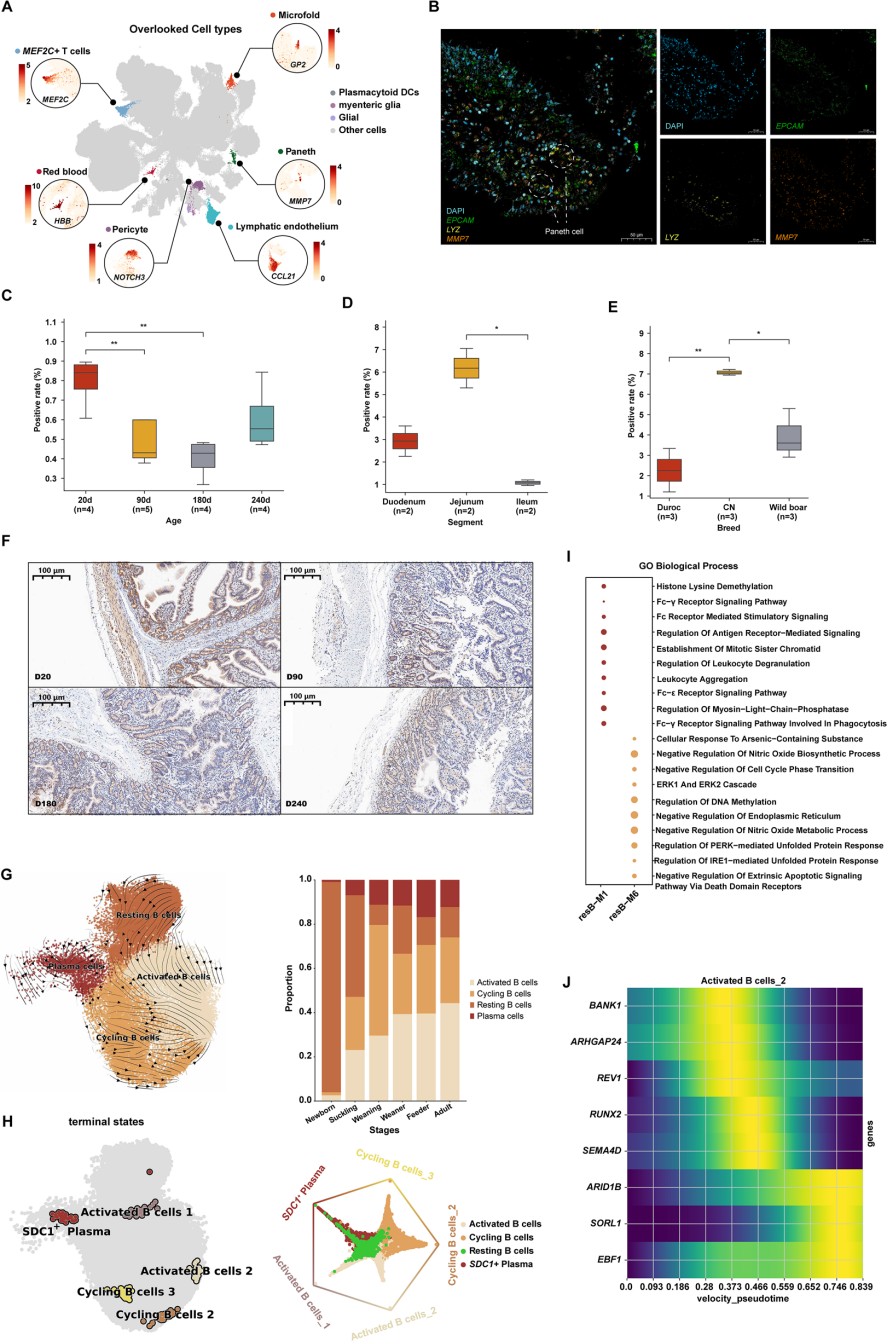
IPGCA recovered previously overlooked cell types and provided biological insights on B cell activation during weaning. A) Cell types that overlooked from original text and their markers. B) Immunofluorescence stained images for Paneth marker, DAPI (blue), *EPCAM* (green), *LYZ* (yellow), and *MMP7* (orange), scale bars = 50 µm. C) Immunohistochemistry positive rate across five ages (20, 90, 180, 240 d) of CN pig jejunum, sample size is listed below. D) Immunohistochemistry positive rate across three segments of intestine (Duodenum, jejunum, ileum). E) Immunohistochemistry positive rate across three breeds, Duroc, CN, and Wild boar. F) Immunohistochemistry stained images along stages (Piglet:20d CN pig, Feeder: 90 d CN pig, Adult: 180 d CN pig, and Adult: 240 d CN pig, scale bars = 100 µm). G) The left figure shows key cell types of B cell lineage with a dashed line and arrows depict paths of differentiation determined by scVelo; The right figure shows cell types of B lineage proportions along developmental stages. H) The left figure shows terminal states of B lineage along pseudotime by CellRank2; The right figure shows circular projection of cells based on fate probabilities. Fate‐biased cells are positioned adjacent to their corresponding fate pole; Naive cells are centralized. Resting B cell clusters are highlighted in green. I) GO enrichment pathway on two core differentially expressed modules identified by hd‐WGCNA of resting B cells. J) The Heatmap of the expression along with velocity pseudotime of core hub genes from the M1 module in “activated B cells_2″ trajectory.

Owing to their transient existence or low abundance, some cell types are often elusive and challenging to identify accurately within individual single cell datasets.^[^ ^37^ ^]^ However, IPGCA has provided us with the capability to identify these previously overlooked cell types effectively (**Figure** **2**A). These cell types include Paneth cell (*n* = 437, *PIGR^high^, MMP7^+^, CPSF6^high^*), Microfold cell (*n* = 803, *EPCAM^high^, GP2^+^*), Plasmacytoid DC (*n* = 105, *ENSSSCG00000010077^+^, VPREB1^+^, JCHAIN^+^, IRF7^high^, MZB1^high^*), Lymphatic endothelium (*n* = 2772, *PROX1^+^, LYVE1^+^, CCL21^+^*), myenteric glia (*n* = 296, *GFAP^+^, CD9^high^*), pericyte (n = 2162, *NOTCH3^+^, MCAM^+^, RGS5^high^, KCNJ8^+^, ABCC9^+^*), red blood cell (*n* = 216, *HBB^+^, HBM^+^*), Glial cell (*n* = 213, *SOX10^high^, GFAP^+^*), and a subset of T cells (*n* = 1590, *MEF2C^+^*, Figure S2A, Supporting Information), characterized by specific features associated with developmental stage and breed, demonstrated an increased prevalence in the small intestine and post‐weaning samples. As red blood cells may represent contamination from the sample environment, we excluded them from subsequent analyses.

Paneth cells, for instance, known for their role in the secretion of antimicrobial peptides (AMPs), and support of intestinal stem cells (ISCs), are regarded as an essential component of intestinal innate immunity.^[^ ^48^ ^]^ However, the presence of Paneth cells in the porcine species, the timing of their appearance, and variations in their abundance remain controversial.^[^ ^48^ ^]^ In the original, individual datasets, Paneth cells were rarely captured. After integration, we successfully annotated a distinct Paneth cell population *(EPCAM^+^*, *LYZ*⁺) and employed immunofluorescence (IF) to validate *MMP7* as a potential Paneth cell marker in swine (Figure 2B; Figure S2B, Supporting Information). Cross‐species comparison revealed high transcriptional similarity between porcine, human, and murine Paneth cells (Figure S2C, Supporting Information).

We next examined Paneth cell abundance across four developmental stages (from piglet to adult), three breeds (Duroc, CN, Wild boar), and three small‐intestinal segments (duodenum, jejunum, ileum). Paneth cell frequencies decreased progressively from piglet to adult 180d, followed by a rebound at 240d (Figure 2C,F). Cells were present in both duodenum and jejunum, but were virtually absent from the ileum (Figure 2D), consistent with previous porcine reports.^[^ ^48^ ^]^ Furthermore, marked inter‐breed differences were observed, with CN pigs exhibiting significantly higher Paneth cell abundance than the other breeds examined (Figure 2E). We also performed cross‐population composite likelihood ratio (XP‐CLR),^[^ ^49^ ^]^ eigenGWAS,^[^ ^50^ ^]^ and *F*_ST_^[^ ^51^ ^]^ analyses between CN pigs (*n* = 228) and Wild boar (*n* = 125) based on genotypic dataset from Pig Haplotype Reference Panel^[^ ^52^ ^]^ (PHARP) (Experimental Section; Table S4, Supporting Information), and defined genomic regions as being under selection only when all three metrics exceeded the top 1% threshold (**Extend** Figure 2E, Supporting Information). Hypergeometric testing against the universe of all cell type DEGs (*n* = 3258) revealed an enrichment of these sweep‐associated genes (*n* = 26) within Paneth cell DEGs (*p* value = 0.037; odds ratio = 1.61, **Extend** Figure 2E, Supporting Information). Among them are key regulators of Paneth cell genesis and function, including the cytoskeletal component *KRT18*, the lineage‐defining transcription factor *SOX9*,^[^ ^53^ ^]^ and *XBP1*, whose ER‐stress‐mediated signaling is essential for maintaining Paneth cell abundance and antimicrobial activity.^[^ ^54^ ^]^ Briefly, our results suggest that Paneth cells exhibit distribution and functional differences across various small intestinal segment, multi‐ages and breeds in pigs.

The IPGCA extend framework enables continuous enrichment of previously overlooked cell types through streamlined data integration. The consolidated IPGCA All now incorporates 9213 Paneth cells, 322 plasmacytoid DCs, 8425 lymphatic endothelial cells, 1095 myenteric glia, 4781 pericytes, 865 glial cells, and 2616 *MEF2C*⁺ T cells.

### 2.4 IPGCA Provide Biological Insights Into B Cell Activation During Weaning

As a comprehensive integrated resource, IPGCA provides a versatile platform for biological investigations. For instance, during the weaning period from 21 to 35 days, piglets frequently encounter gastrointestinal cell‐mediated hypersensitivity, leading to intestinal injury and diarrhea as they establish immune tolerance to dietary antigens. During the early postnatal period from newborn to weaning stage a pronounced trend in B cell differentiation was observed, with a decrease in resting B cells and a concurrent rise in cycling B cells, activated B cells, and plasma cells (Figure 2G; Figure S2D, Supporting Information). To confirm the proportion change, we utilized a permutation test to calculate *p* value of the difference, and we found that weaning state significantly increases cycling B cells and decreases resting B cells compared to suckling piglets (Figure S2E, Supporting Information), while the weaner pig significantly increases resting B cells than weaning state. Trajectory and RNA velocity analysis^[^ ^55^ ^]^ consistently reveal a cellular transition process including reactivation from memory B cells or an initial activation event by naïve B cells, resulting in resting B cells toward activated B cells, cycling B cells and Plasma cells (Figure 2G)

We then leveraged CellRank2 based on gene‐expression similarity and RNA velocity to further characterize this activation trajectory. Five distinct terminal states were defined, and fate probabilities for every cell were computed. Resting B cells emerged as the sole progenitor state, giving rise to cycling B, activated B and plasma cell fates with high clarity (Figure 2H). Notably, the plasma‐cell trajectory resolved into a specialized plasma subset (*SDC1*⁺/*CD138*⁺ plasma) (Figure 2H; Figure S2F, Supporting Information), mirroring the post‐weaning B cell transition reported by Tang et al.^[^ ^56^ ^]^ To capture the global regulatory landscape underlying resting B cell activation at weaning stage, we applied high‐definition weighted gene co‐expression network analysis (hd‐WGCNA),^[^ ^57^ ^]^ and discerned six distinct gene modules between weaning piglet and suckling piglet, labeled M1 to M6 (Figure S2G, Supporting Information), within the quiescent B cell subset. Analysis of the dynamic changes in module expression (DME) revealed that modules M1 and M6 exhibited significant temporal associations. Subsequent enrichment analysis of these modules indicated that M1 was enriched in pathways such as “Fcγ Receptor Signaling Pathway (GO:0038094)” and “Leukocyte Aggregation” (GO:0070486), signifying its pivotal role in immune response activation (Figure 2I). Module M6, on the other hand, was enriched in pathways including “Cellular Response to Arsenic‐Containing Substance (GO:0071243)”, “ERK1 and ERK2 Cascade (GO:0070371)” and “Negative Regulation of Cell Cycle Phase Transition (GO:1901988)”, suggesting its involvement in cell proliferation and differentiation in stress response.

Moreover, we weighted each cell's contribution to each trajectory by its fate probability and visualized the expression trajectories of lineage‐specific genes. Within the M1 module, genes orchestrate the velocity pseudotime trajectory: early BCR receptor signaling *BANK1* ^[^ ^58^ ^]^ and Rho‐GTPase‐mediated cytoskeletal remodeling *ARHGAP24* ^[^ ^59^ ^]^ expression at the onset, followed by the early B cell factor *EBF1*,^[^ ^60^ ^]^ and *RUNX2–KLF2* interactions^[^ ^61^ ^]^ that consolidate differentiation (Figure 2J). In the M6 module, we observed early‐velocity pseudotime up‐regulation of multiple genes and transcription factors governing B‐cell proliferation and differentiation, including *BACH2*,^[^ ^62^ ^]^ *ID2*,^[^ ^63^ ^]^ *MYC*,^[^ ^64^ ^]^ *ODC1*,^[^ ^65^ ^]^ *ZFP36L2* ^[^ ^66^ ^]^ and *HERPUD1*,^[^ ^67^ ^]^ an ER‐stress marker that modulates plasma cell commitment (Figure S2H, Supporting Information). These observations are in line with the immunological responses observed in weaning pigs, where dietary shifts trigger the activation of resting B cells, prompting their transition into cycling B cells, activated B cells and plasma cells, which in turn bolster the adaptive immune response.

### 2.5 IPGCA Provides Cellular Insight of Large‐Scale Genetic Studies of Pig Complex Traits

Compared with bulk RNA‐seq data, the IPGCA can provide cellular context to identify traits‐critical cell types in combination with GWAS results. Several GWAS studies have been conducted on economic traits and growth‐related traits in pigs, identifying numerous genetic variants associated with these traits. However, the mechanisms by which these genetic variants influence specific tissues and cell types remain unknown. To address this, we analyzed 232 GWAS summary statistics from PigBiobank^[^ ^68^ ^]^ (Table S5, Supporting Information) in conjunction with bulk tissues and IPGCA using scDRS,^[^ ^20^ ^]^ by evaluating polygenic trait/disease enrichment score of tissues and cells.

**Figure 3:**
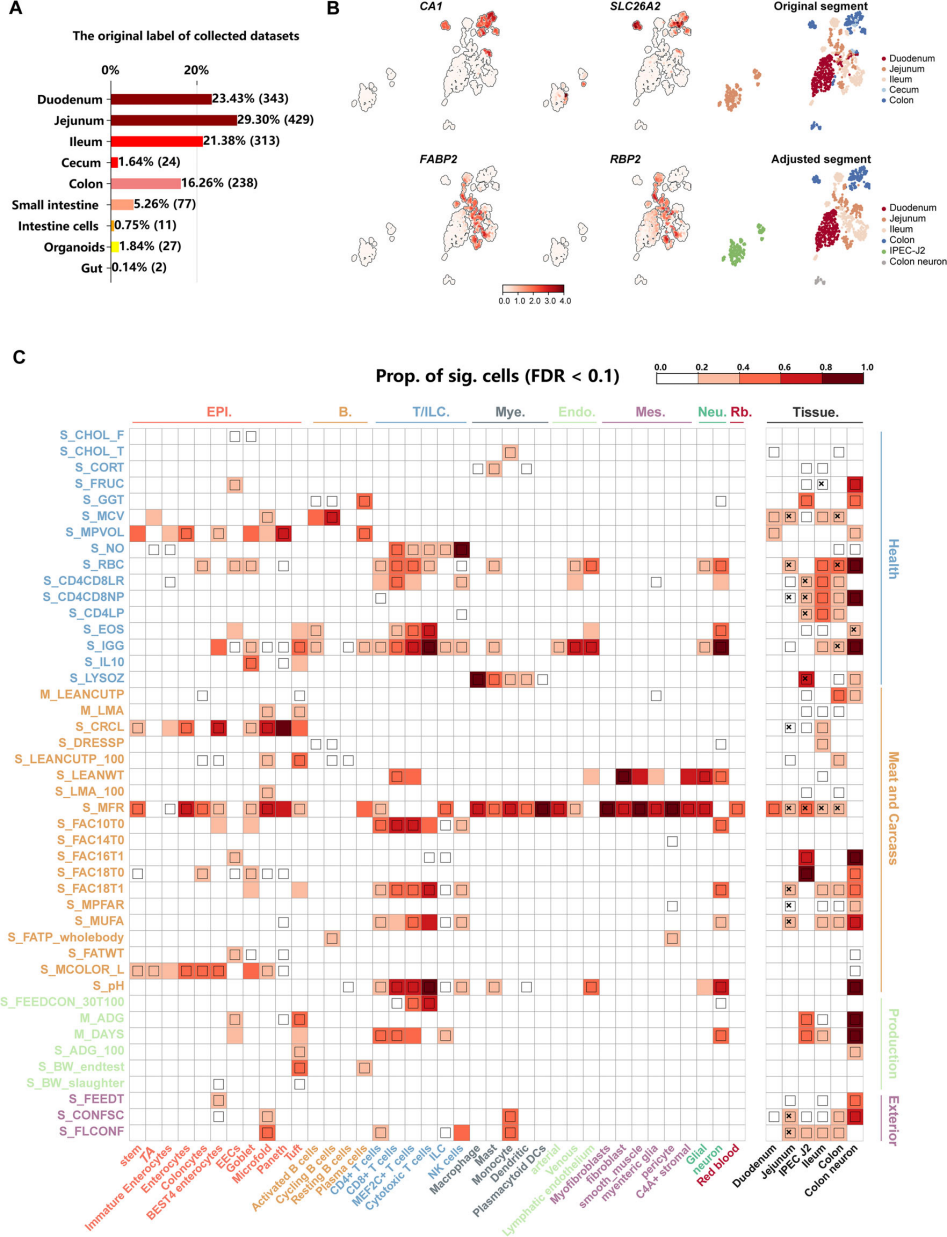
Mapping complex traits into IPGCA. A) Summary statistics of the 1464 collected public RNA‐seq datasets, categorized by intestinal segment (jejunum, duodenum, ileum, colon, and cecum). B) Feature map of marker genes specific to the small and large intestines (*FABP2*, *RBP2* for the small intestine; *CA1*, *SLC26A2* for the large intestine), along with a comparison of label corrections before and after clustering. C) Enrichment results of scDRS for 38 cell types and a subset of 44 complex traits, which showed at least one association from PigBiobank. Each row represents a complex trait (with colors indicating different trait categories For detailed traits names corresponding to abbreviations, see Table S5, Supporting Information) and each column represents a cell type (with colors indicating cell lineages). Heatmap cell colors for each cell type‐trait pair denote the proportion of significantly associated cells (“Prop. of sig. cells” in the figure legend; FDR < 0.1 across all cells for a given trait). Squares within cells denote significant cell type‐trait associations (FDR < 0.05 across all pairs). Cross symbols denote significant heterogeneity in association with traits across individual cells within a given cell type (FDR < 0.05 across all cell type‐trait pairs).

To explore the enrichment association in tissue level, we collected 1480 public porcine intestinal RNA‐sequencing datasets (Table S6, Supporting Information), covering jejunal (*n* = 429, 29.3%), duodenal (*n* = 343, 23.43%), ileal (*n* = 313, 21.38%), colonic (*n* = 238, 16.26%), and cecal (*n* = 24, 1.64%) segments (**Figure** **3**A). Given the potential for labeling errors or inconsistencies in spatial positioning during intestinal sample collection, we clustered the samples using uniform manifold approximation and projection (UMAP) based on their expression levels and refined the clustering using the Leiden algorithm. We visualized the expression of marker genes specific to the small and large intestines (*FABP2*, *RBP2* for the small intestine, and *CA1*, *SLC26A2* for the large intestine, Figure 3B). We excluded outlier samples (*n* = 16), corrected evident mislabeling in some clusters, and categorized the remaining tissues into seven distinct groups, including duodenum, jejunum, ileum, colon, IPEC‐J2 (porcine enterocytes isolated from the jejunum of a neonatal piglet), and two combined groups for small and large intestines. In addition, 35 samples were identified as neuronal large intestine samples (Figure 3B). For single cell level, the enrichment association were conducted based on IPGCA.

Among 232 traits, 62 traits showed association with at least one intestinal segment/cell line (Figure S3A, Supporting Information), and 44 traits exhibited associations with at least one cell type of IPGCA (Figure 3C). We categorized these traits into health traits (e.g., blood parameters and immune capacity), meat and carcass traits (e.g., anatomy and fatty acid content), production traits (e.g., growth and feed conversion), and exterior traits (e.g., conformation). We identified a total of 529 associated tissue‐trait/cell type‐trait pairs (FDR < 0.1) (Figure 3C). Some tissue‐level associations were further enriched at the cell type level. For example, S_LYSOZ (meta‐GWAS for lysozyme level) was enriched in colon tissue and was further enriched in the myeloid lineage in IPGCA (Figure 3C). M_ADG (meta‐GWAS for average daily gain) was enriched in ileum and further exhibited the association with enteroendocrine cells (EECs), tuft cell and Paneth cell (Figure S3D, Supporting Information). Conversely, there were associations at the tissue level that were not identified at the cell type level, such as M_BFT (meta‐GWAS for backfat thickness), which showed associations across most of the gut tissues (Figure S3A, Supporting Information). At the cell type level, notably, health traits exhibited enrichment mainly in the T/ILC lineage, endothelial lineage, and specific epithelium (Figure 3C). Blood biochemical indices (such as CD4^+^/CD8^+^ leukocyte ratio, CD4^+^, CD8^+^ leukocyte percentage, eosinophil number, immunoglobulin G level) also correlated with neuron cell types. Meat and carcass traits were associated with a diverse range of epithelial cell types, and exhibited an enrichment within the T/ILC cell categories and mesenchymal cells. Production traits were linked to EECs, tuft cells, and multiple T cell sub types. For instance, M_LMA (meta‐GWAS for loin muscle area) was associated with microfold and tuft cells (Figure S3B, Supporting Information). Exterior traits were enriched in *BEST4* enterocytes, microfold, and some immune cell types. Consequently, by linking GWAS signals to intestinal cell types, the IPGCA serves as a potential valuable resource to enhance our understanding of intestinal function and its correlation with complex traits.

### 2.6 IPGCA Acts As a Reference for Cell Type Deconvolution of Bulk RNA

**Figure 4:**
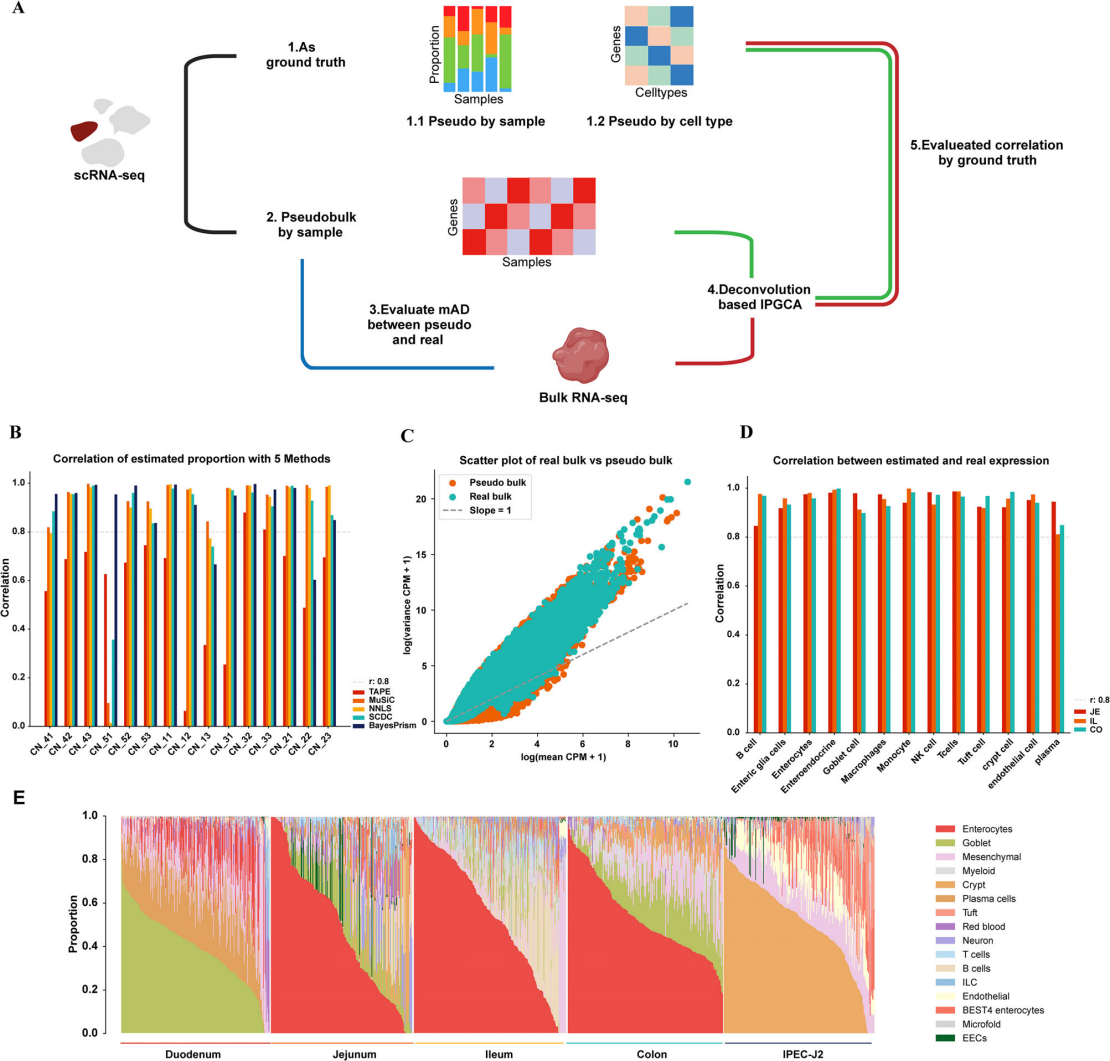
Benchmark of deconvolution process and collection of bulk RNA‐seq. A) Flowchart of the benchmarking process for deconvolution, the scRNA‐seq data were subjected to two types of processing: 1) serving as ground truths for generating pseudo‐bulk data by sample and by cell type, and 2) using pseudo‐bulk data by sample as the deconvolution test samples (indicated by the black line). The entire testing process is illustrated by three lines: the green line represents deconvolution of single‐cell data into pseudo‐bulk by sample to obtain the estimated composition compared to the ground truths; the red line shows deconvolution of bulk RNA‐seq data to obtain estimations compared to the ground truths; and the blue line indicates a comparison between pseudo‐bulk data by sample and bulk RNA‐seq data. B) Performance of deconvolutional cell proportion estimation using pseudo‐bulk samples for five different methods, evaluated by Pearson's correlation coefficient. C) Scatter plot comparing the mean and variance of expression data from deconvolution results and matched real bulk data. D) Performance assessment of the deconvolutional expression matrix derived from pseudo‐bulk samples using BayesPrism, with the expression matrix from pseudo‐bulk samples by cell type serving as the ground truth. E) Deconvolution results of public bulk RNA‐seq datasets, showing the distribution patterns of cell types across different intestinal segments.

To test whether IPGCA can be used as a reference for cell type deconvolution of bulk RNA‐seq data, we selected 15 scRNA‐seq samples that matched corresponding bulk RNA‐seq data to evaluate the performances of five different deconvolution methods. The cell type proportions and expression matrix derived from scRNA‐seq were used as ground truths. We first generated pseudo‐bulk data from these 15 scRNA‐seq samples and deconvoluted them to estimate cellular composition based on IPGCA (excluding these samples). The accuracy of deconvolution was assessed using Pearson's correlation coefficient between estimation and ground truths (**Figure** **4**A). Among the 5 deconvolution methods, BayesPrism^[^ ^23^ ^]^ demonstrated superior performance, achieving a mean correlation of 0.91 ± 0.12 for cellular expression across diverse samples. In contrast, the remaining methods exhibited diminished correlations under specific conditions (Figure 4B). Additionally, the cell type × gene expression profile obtained from BayesPrism showed a high correlation with the expression matrix from pseudo‐bulk data generated by major cell types (Figure 4D; Experimental Section).

We further tested the similarity between real bulk of these samples and single cell datasets using mean absolute deviation (mAD) (Figure 4C). Though the correlation of deconvolutional expression based on pseudo‐bulk samples exhibited a good performance, the deconvolution result based on real bulk sample varied among cell types. Specifically, some cell types, such as EECs and plasma cells showed low accuracies and were thus excluded from subsequent analysis (**Extend** Figure 3C, Supporting Information). Overall, these results affirmed the effectiveness of BayesPrism in the deconvolution process applied to bulk samples.

We then conducted deconvolution using BayesPrism to estimate the posterior means of cell type proportions and gene expression levels across all samples. We observed distinct patterns in the distribution of cell types across intestinal segments (Figure 4G). In the duodenum, goblet, plasma, mesenchymal, and enterocyte cell types were notably more prevalent. In contrast, the jejunum was characterized by a near 50% prevalence of enterocytes. The ileum exhibited a continued high prevalence of enterocytes, alongside an increase in immune cells. In the colon, enterocytes and goblet cells were the predominant cell types. For the IPEC‐J2 cell type, originating from jejunal cells of a neonatal unsuckled piglet, the cells predominantly consisted of juvenile enterocytes that share transcriptional similarities with crypt cells. Future transcriptomic data can be continuously deconvoluted based on the IPGCA to obtain results at the cell type proportion level, facilitating the investigation of cellular differences.

Given the performance of our deconvolution approach, we demonstrate its utility in scDRS association analyses using in‐house RNA‐seq datasets from two genetically distinct Duroc lines (D1: *n* = 42; D2: *n* = 55) exhibiting divergent loin muscle area (LMA). The D1 and D2 lines are two genetically differentiated populations derived from long‐term, divergent selection. Notably, D2 exhibits a high‐backfat, low‐loin‐muscle‐area phenotype (Figure S3E and Table S7, Supporting Information). We employed deconvolution analysis to investigate the differential expression of M_LMA‐associated top genes identified by MAGMA^[^ ^69^ ^]^ in key cell types (identified by scDRS) between D1 and D2 and revealed that the GWAS candidate genes *ALR15*, *FKBP5*, and *HMGA1* are differentially expressed in tuft and microfold cells within the D1 and D2 populations (Figure S3F, Supporting Information). These genes are all associated with glucose metabolism and insulin sensitivity. Given that tuft cells serve as chemosensors in the intestine, their differential expression patterns suggest a role in metabolic processes and muscle development.^[^ ^70^, ^71^, ^72^ ^]^ Further enrichment results indicated these top genes were also related to the “Glycerophospholipid metabolism (ssa00564)” pathway **(Extended** Figure 3E, Supporting Information), but these correlations await validation through complementary multi‐omics datasets and targeted experiments.

### 2.7 Combine IPGCA with RNA‐Seq to Identify Cell Type Specific Genetic Regulatory Effects

To investigate the genetic regulatory influences on gene expression across various cell types, we performed single nucleotide polymorphism (SNP) calling using a bulk RNA‐seq data analysis pipeline, as described by PigGTEx.^[^ ^11^ ^]^ The accuracy of genotyping was assessed utilizing duodenum samples (*n* = 300),^[^ ^6^ ^]^ which supplied both RNA‐seq and high‐coverage (>10×) whole genome sequencing (WGS) data. After imputation based on haplotype reference panel from PGRP,^[^ ^11^ ^]^ the concordance rate (CR) between genotypes identified by RNA‐seq and WGS reached 0.91 ± 0.02 (Figure S4A, Supporting Information).The imputation confidence score (DR2) exhibited higher values in regions near transcription start sites (Figure S4B, Supporting Information), thereby validating the use of sites within 1 Mb upstream and downstream of genes for subsequent cis‐eQTL mapping.

We first identified cis‐eQTLs at the tissue level, with sample sizes ranging from 192 (ileum) to 799 (small intestine) individuals (Figure S4D, Supporting Information). This analysis took into account potential confounding factors by PEER^[^ ^73^ ^]^ (10 PCs and 10 peer factors, see methods) and controlled for multiple testing. A gene was considered as an eGene for a specific tissue if it possessed at least one significant cis‐eQTL. The number of eGenes discovered ranged from 5453 in the ileum to 15987 in the entire small intestine. Consistent with previous research, the detection rate of eGenes was positively associated with sample size (*r* = 0.768, *p* value = 4.36 × 10^−2^). With an increased sample size, we identified numerous eGenes and eQTLs of intestinal tissues that were previously undetected (Figure S4E, Supporting Information).

**Figure 5:**
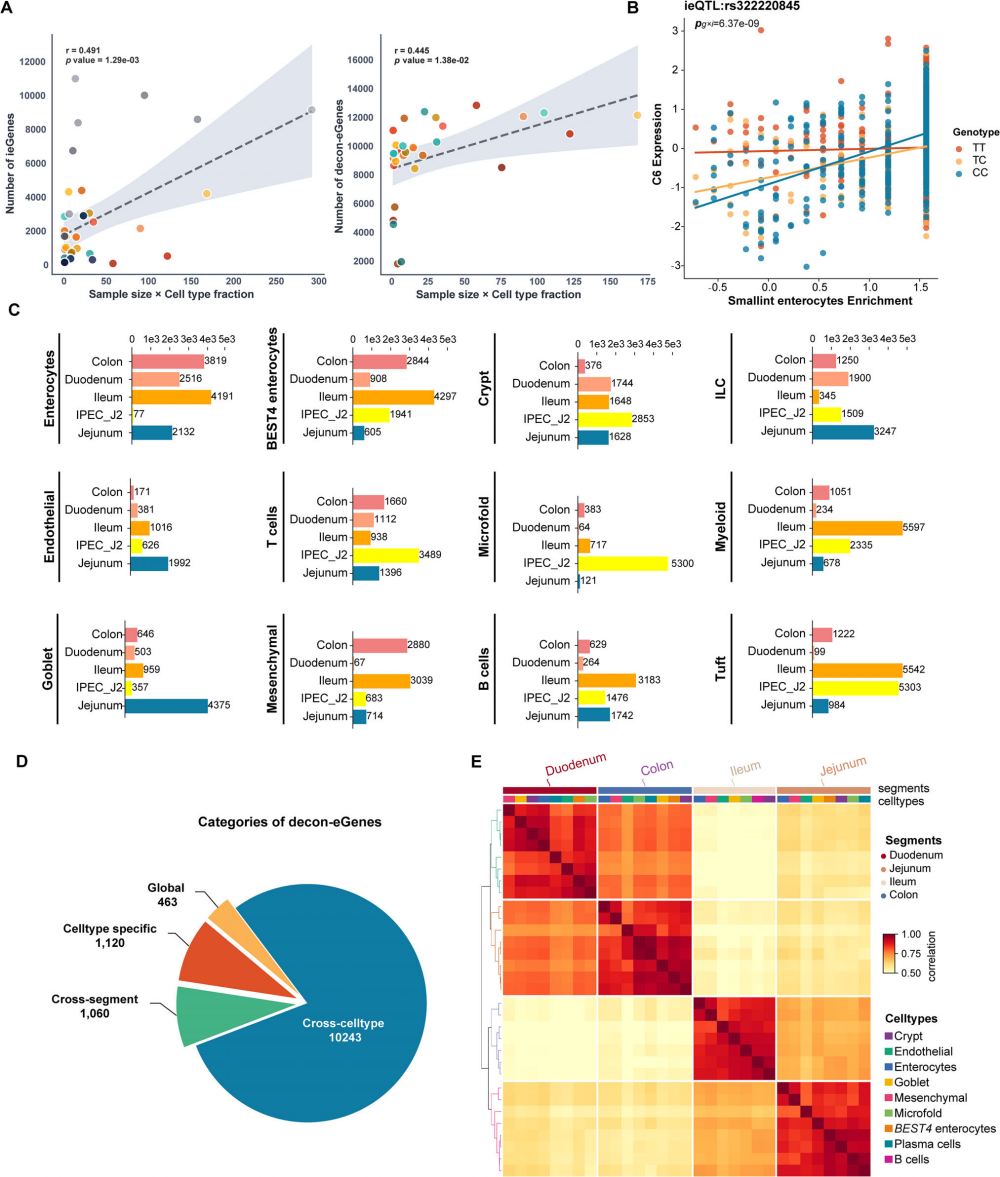
Identification of cell type related cis‐eQTLs. A) Scatter plots of eGene counts across cell type–tissue pairs along with sample size×fraction; (left: ct‐ieGenes, right: decon‐eGenes). B) Effect of eVariant (rs322220845) of *C6* interacted with enterocytes enrichment in the small intestine. C) Number of ct‐ieQTLs discovered in each cell type‐tissue combination at FDR < 0.05. D) Categories of decon‐eGenes: found in one cell type–tissue pair (decon‐ct‐eGenes); found in one cell type across different intestinal segments (cross‐segment decon‐eGenes); found in multiple cell types (cross‐cell type decon‐eGenes); found in all nine major cell types (global decon‐eGenes) and their respective numbers. E) Percentage of top eQTLs shared between two cell types. Top eQTLs are considered shared if they are significant in both cell types (LFSR ≤ 0.05) and the mashR‐estimated effect size is within a factor of 0.1. Cell types are annotated above according to lineage and major cell type. The median pairwise percentage sharing per lineage is shown in black.

We then focused on the specificity of intestinal cell types and identified two distinct categories of cell type cis‐eQTLs. The first category utilized the variability in deconvoluted cellular composition to assess the genetic interaction with cell types, termed cell type interaction cis‐eQTLs (ct‐ieQTLs). The second category employed the deconvoluted gene expression profiles of individual cell types as phenotypes for the identification of deconvolution‐based cis‐eQTLs (decon‐eQTLs). Both the number of ieGenes and decon‐eGenes correlate positively with the product of sample size and cell type proportion (ieGene: *r* = 0.49, *p* value = 1.29 × 10^−3^; decon‐eGene: *r* = 0.45, *p* value = 1.38 × 10^−2^, **Figure** **5**A).

For ct‐ieQTL, genes whose expression levels were significantly impacted by at least one cell type interaction cis‐eQTL (ct‐ieQTL, FDR<0.05) were identified as ieGenes, with their numbers ranging from 64 (duodenum microfold) to 5597 (ileum myeloid) (Figure 5C; Figure S4F, Supporting Information). The ileum had the highest number of ieQTLs among the intestinal segments (Figure 5C). There was significant variation in the distribution of ieQTLs across different segments for the same cell type. For instance, the microfold cells in the IPEC‐J2 cell line had 5300 ieGenes, while other segments show lower levels (Figure 5C). Additionally, variants related to goblet cells were enriched in the jejunum, and enterocytes exhibited a higher number of ieGenes across all segments but were less abundant in the IPEC‐J2 cell line (Figure 5C). This discrepancy may be due to differences in expression patterns between the epithelial cells of the cell line and those of the sampled tissues, as the deconvolution cell proportion results indicated that cells in IPEC‐J2 may be more similar to crypt cells (Figure 4E). Consequently, a higher number of ieGenes are detected in crypt cells. The gene *C6*, which is associated with inflammatory bowel disease (IBD) in human datasets, was further characterized in our results as interacting with small intestine enterocytes (Figure 5B).

For decon‐eQTL mapping, Genes identified with at least one decon‐eQTL were defined as decon‐eGenes. We identified a total of 12886 decon‐eGenes (Figure S4G, Supporting Information), of which 1120 were cell type specific eGenes (decon‐ct‐eQTL). Additionally, 1060 eGenes were found to be specific to the same cell type across various intestinal segments and were categorized as cross‐tissue decon‐eGenes, accounting for 8.23% of the identified genes. A subset of 463 eGenes was observed in all nine major cell types and was labeled as global decon‐eGenes, constituting 3.59% of the total. The remaining 10243 were categorized as cross‐cell type decon‐eGenes (Figure 5D). We employed mashR^[^ ^74^ ^]^ (multivariate adaptive shrinkage) to discern the patterns of eQTL sharing and specificity (eQTLs were deemed significant with a local false sign rate (LFSR) threshold of 0.05 in at least one cell type and 0.1 in any additional cell type). Our findings indicated a substantial level of eQTL sharing across all cell types, with a mean sharing rate of 0.68. Notably, cells from the same intestinal segment showed a higher concordance. The eQTL sharing rates were notably high, reaching 90.5% in the colon, 89.4% in the duodenum, 89.8% in the jejunum, and 91.3% in the ileum (Figure 5E). Additionally, a significant degree of eQTL sharing was observed between different intestinal segments, particularly between the jejunum and ileum, as well as between the duodenum and colon (Figure 5E).

### 2.8 Systematic Cell Type Resolved Co‐Localization Events With Pig Complex Traits

To evaluate the application of cell type eQTLs in dissecting the genetic mechanisms of complex traits, we performed co‐localization analysis between cell type cis‐eQTLs and meta‐GWAS summary statistics for 144 complex traits from PigBiobank^[^ ^68^ ^]^ (exclude the reproduction traits from 232 PigBiobank traits). We identified a total of 4933 significant co‐localization events (posterior probability of co‐localization, PPH4 > 0.9) within the intestinal tract (Figure S4H, Supporting Information). The ileum exhibited the most co‐localization events (*n* = 1849), with cell types such as mesenchymal (*n* = 1084), enterocyte (*n* = 994), endothelial (*n* = 771), goblet (*n* = 706), crypt (*n* = 590) and microfold cells (*n* = 346) showing a substantial number of co‐localizations (Table S8, Supporting Information). Among the complex traits, significant co‐localization events were primarily observed for adipose deposition traits, such as muscle fat ratio (S_MFR), loin muscle area (M_LMA), and backfat thickness (M_BFT), as well as carcass traits such as carcass length (S_CRCL). Furthermore, genes including *NDUFB2, MS4A12, FAM78A, CPSF7, BMP6, ARL10, APMAP*, and *ABCD4* were identified as having the most co‐localization occurrences, highlighting their potential roles in gut‐related traits (Figure S4H, Supporting Information).

Furthermore, SNP level co‐localization events were characterized by GWAS and eQTL *p* values less than 5×10^−8^ and PPH4 > 0.9. These events were concentrated on traits such as S_MFR, with the *MUC2* gene being notably enriched. *MUC2*, a sub‐type of secretory mucins produced by goblet cells, is the primary macro‐molecular component of mucus.^[^ ^75^ ^]^ This finding suggests that economic traits like S_MFR are inextricably linked to the expression regulation of the intestinal mucosa and goblet cells. A previous study has shown that GWAS loci associated with backfat thickness co‐localized with cis‐eQTLs of *ABCD4* in both the brain and small intestine tissues.^[^ ^11^ ^]^ Our study further identified this co‐localization in specific cell types including enterocytes, mesenchymal cells, and microfold cells, particularly in the ileum (Figure S4H and Table S8, Supporting Information). This indicates that backfat thickness may be influenced by adipose deposition under the joint regulation of different intestinal cell types.

### 2.9 Divergent Co‐Localizations Across Three eQTL Modalities With Fine‐Mapped GWAS Loci

**Figure 6:**
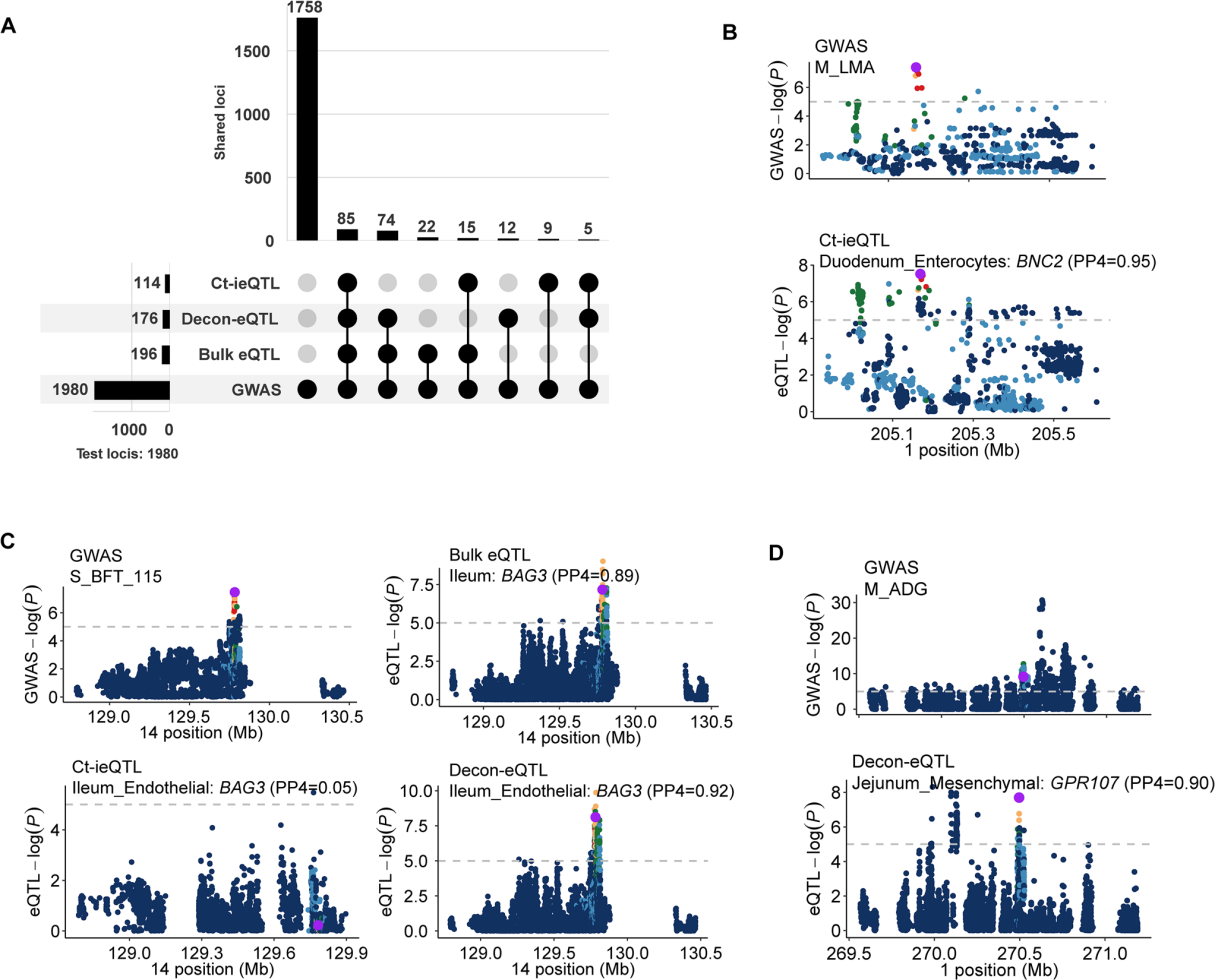
Co‐localization between ct‐eQTL and pig complex traits GWAS signals. A) The plot shows the count of GWAS independent loci that co‐localized with each eQTL class (bulk eQTL, ct‐ieQTL, and decon‐eQTL). B) The case of co‐localization between GWAS of M_LMA and ct‐ieQTL of *BNC2* at duodenum enterocytes on chromosome 1 (rs332553917). C) The co‐localization case between GWAS of S_BFT_115 GWAS (left top) and bulk eQTL (right top), ct‐ieQTL (left bottom), decon‐eQTL (right bottom) of *BAG3* on chromosome 14 (rs328847863). D) The co‐localization case of GWAS of M_ADG and decon‐eQTL of *GPR107* at jejunum mesenchymal on chromosome 1 (rs323809827).

To pinpoint biologically meaningful regulatory signals, we further fine‐mapped 1980 independent loci from the meta‐GWAS (Experimental Section) and then performed co‐localization analyses between these independent loci and three types of eQTLs (bulk eQTLs, ct‐ieQTLs, and decon‐eQTLs). Global co‐localization patterns revealed bulk eQTLs colocalized with 198 loci, decon‐eQTLs with 176, and ct‐ieQTLs with 114 (**Figure** **6**A). The majority of these co‐localizations were shared among the eQTL types; only 22, 12, and 9 loci were uniquely colocalized with bulk‐, decon‐, and ct‐ieQTLs, respectively (Figure 6A). Building on this, we uncovered numerous eQTL signals that were masked in bulk datasets became detectable at cellular resolution or signals in bulk were further resolved at the single cell level. For instance, while bulk eQTLs showed no co‐localization between M_LMA and *BNC2* in duodenum, rs332553917 exhibited strong co‐localization (PPH4 = 0.99) with enterocyte‐specific ieQTLs (Figure 6B). *BNC2* has previously been implicated in neuronal circuits that acutely suppress appetite and in myofibroblast activation.^[^ ^76^ ^]^ These findings suggested that these appetite‐ and muscle‐activating roles may be functional within intestinal enterocytes and ultimately influenced loin‐muscle‐area phenotypes.

Another case showed the decon‐eQTLs captured a higher resolution regulator mechanism. we identified *BAG3* as a colocalized gene in the ileum via bulk eQTL analysis. Decon‐eQTL analysis further localized *BAG3* to endothelial cells in the ileum, while ct‐ieQTL analysis did not detect co‐localization (Figure 6C). *BAG3* is commonly associated with atherosclerosis, which is characterized by the abnormal accumulation of lipids^[^ ^77^ ^]^ (such as cholesterol and triglycerides) in the vascular wall. This process involves the loss of characteristic endothelial cell markers (e.g., *CD31*) and their subsequent transformation into mesenchymal cells. These findings provide insights into the mechanisms underlying backfat deposition in pigs. Similarly, bulk‐eQTL resolved *GPR107* co‐localization in jejunuml cells (PPH4 = 0.94) for average daily gain (ADG), whereas decon‐eQTL analysis showed further association in jejunuml cells (PPH4 = 0.90, Figure 6D). These cases elucidate that the specific regulatory mechanisms of GWAS loci may occur within cell types that may be obscured in the resolution of bulk eQTLs.

### 2.10 Leveraging IPGCA for Mapping Human Complex Traits

**Figure 7:**
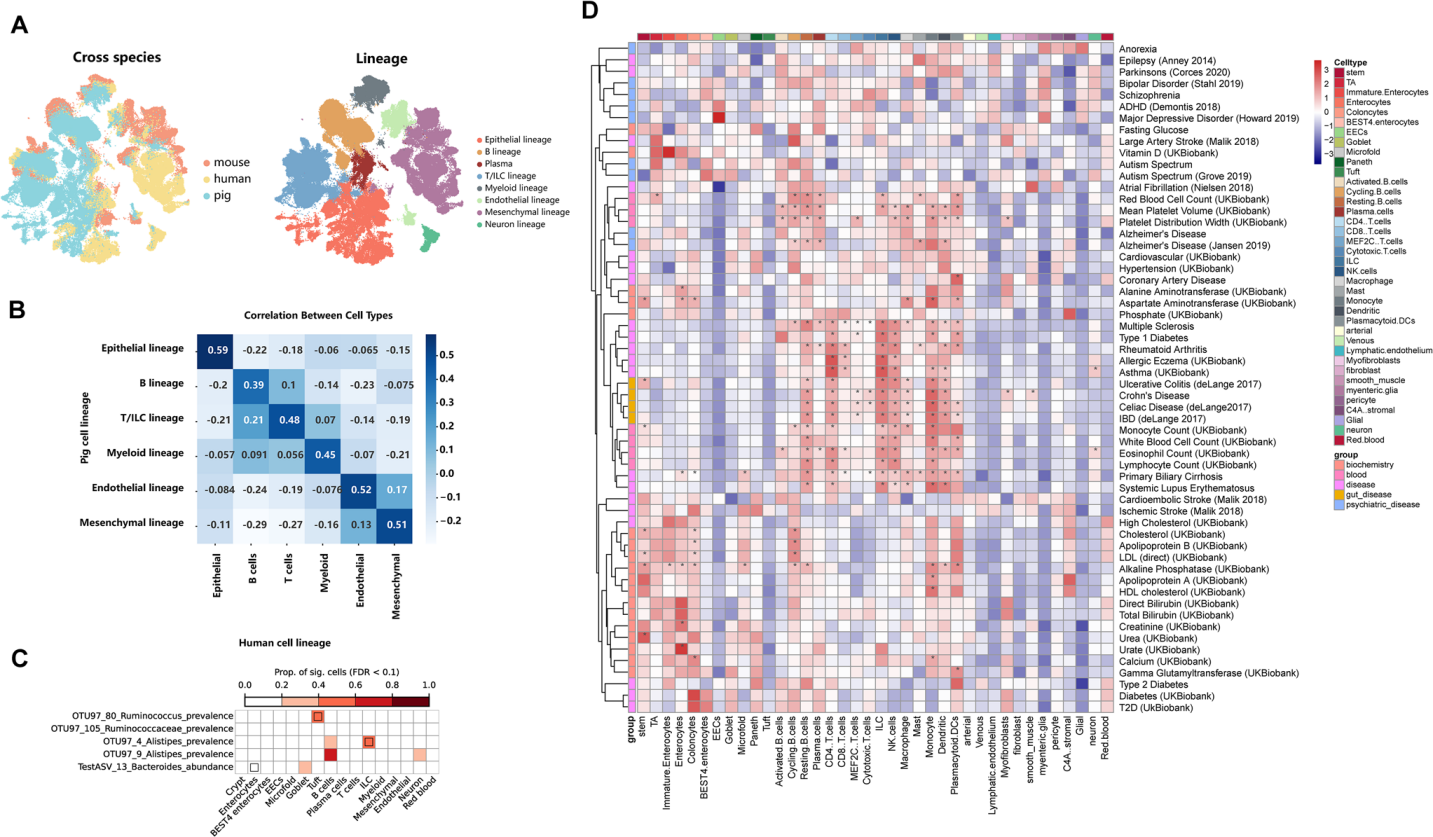
Leveraging IPGCA for mapping human complex traits. A) Integrated atlas of human, mouse, and pig in UMAP in terms of species and lineages. B) The Pearson's correlation coefficients of gene expression between human and pig cell types. C) Five microbial taxa were significantly enriched in specific cell types. D) The heatmap displays the heritability enrichment score of 176 human complex traits/diseases from the UK Biobank in various gut cell types derived from IPGCA.

Extensive evidence has demonstrated that pigs are excellent model organisms for studying human physiology,^[^ ^3^, ^4^, ^78^ ^]^ and pigs and humans share a high degree of physiological and anatomical similarity. While prior studies have explored human‐pig transcriptional similarities at cellular resolution,^[^ ^33^, ^44^ ^]^ our work advances this paradigm through systematic assessment of intestinal cellular architecture conservation and molecular divergence between species, and further evaluates porcine intestine as a tractable model for dissecting human complex traits. We first integrated human single cell data from the Gut Cell Survey^[^ ^31^ ^]^ (https://www.gutcellatlas.org/) with IPGCA, as well as a mouse dataset^[^ ^79^ ^]^ from GSE264408 (**Figure** **7**A). We then calculated the Pearson's correlation coefficients for global gene expression patterns across human, murine, and porcine cell lineages (Figure 7B; Figure S5A, Supporting Information). Notably, excluding the B lineage (where the IPGCA included plasma cells, unlike the human reference atlas, which classified them distinctly), the porcine data showed a more pronounced correlation with the human dataset compared to the murine dataset. We further compared the correlation between DEGs for each cell type (Experimental Section), revealing that pigs show greater similarity with humans in terms of cell type expression level (pig: 0.85 ± 0.05; mouse: 0.81 ± 0.09, Figure S5C,D, Supporting Information). These findings enabled us to link human complex‐trait associations to porcine atlas based on expression correlations, thereby providing cell type level mechanistic insights.

Therefore, we further mapped public human microbiome‐wide association studies (mGWAS) summary statistics to IPGCA using scDRS to identify potential associated cell types for microbiota. Among 100 gut microbiota prevalence GWAS summary statistics, five microbial taxa were significantly associated with different cell types (Figure 7C). These findings were consistent with the conclusions from previous studies. For instance, we found that Bacteroides was enriched in enterocytes and goblet cells. A previous study by Wang et al^[^ ^80^ ^]^ identified that Bacteroides fosters the differentiation of goblet cells by producing propionate and Laura Wrzosek et al^[^ ^81^ ^]^ (2013) found that acetate produced by Bacteroides up‐regulated *KLF4*, a transcription factor involved in goblet cell differentiation. These results indicated the complex interplay and regulatory mechanisms between the gut microbiota, goblet cells, and the mucus layer.

In addition to scDRS, we employed the heritability enrichment to cell types by LDSC‐seg across 176 diverse diseases and traits related to gut (Table S8, Supporting Information). The phenotypes most significantly enriched for heritability across diverse cell types are those related to blood and gastrointestinal diseases (Figure S5B, Supporting Information). Intestinal diseases and blood indices are primarily enriched in the T/ILC, B, and myeloid lineages. Various physical and chemical body indices (e.g., bilirubin, trace elements, urates) are enriched in the epithelial and myeloid lineages. Except for Alzheimer's disease, which was significantly enriched in the B and myeloid lineages, other mental disorders did not show significant enrichment in these lineages (Figure 7D). In summary, IPGCA can play a role in exploring the associations between human complex traits/diseases and specific cell types.

### 2.11 Construction of scGutDB

We have developed an online platform named scGutDB (http://alphaindex.zju.edu.cn/scgut) to fully utilize the resources of intestinal single cell data, enabling users to visualize and explore findings in the paper. It provides our comprehensive atlas IPGCA, as well as some human, pig, and mouse intestines scRNA‐seq datasets. The platform also facilitates the sharing analysis pipelines and code, and offers functionalities for query and visualization.

To serve as a reference of further analysis, we utilized the CellTypist^[^ ^25^ ^]^ module to train an automated cell type annotation model. The model's performance was evaluated on a validation dataset to assess the accuracy of the automated annotation. The results of automated annotation were compared with the annotations provided in the original publications, and a strong confidence score was observed at the lineage level (Figure S6A, Supporting Information), indicating robust predictions.

The platform consists of seven main modules: 1) Home page: It provides an overview of the website and showcases the single cell atlases of IPGCA (Figure S6B, Supporting Information). 2) Marker query: It offers queries for the DEGs of IPGCA for each cell type and visualization tools (Figure S6C, Supporting Information). 3) Explore data: scGutDB facilitates interactive exploration of IPGCA and Human Gut Cell Atlas, offering visualization for any gene of interest along with all associated covariates. For those intrigued by dynamic gene expression patterns, both violin plots and dot plots can be generated in conjunction with a selected covariate, providing a comprehensive view of gene expression variation across different biological contexts (Figures S6D and S7E, Supporting Information). 4) eQTL query: This platform enables the interrogation of a comprehensive repository of eQTL data, encompassing eQTLs, ct‐ieQTLs, and decon‐ct‐eQTLs. Users can perform searches using identifiers such as SNP ID, Gene ID, tissue, and cell type, thereby facilitating targeted investigations into the genetic architecture underlying gene expression variation across specified biological contexts (Figure S6F, Supporting Information). 5) Auto annotation: This module employs a large collection of intestinal single cell data to train automatic annotation models (CellTypist.pkl files) for various cell lineages (Figure S6G, Supporting Information). 6) GWAS enrichment: This module shares the process of enrichment between intestinal cell types and relevant GWAS results using software such as MAGMA and scDRS, and performs heritability enrichment analysis with LDSC‐seg (Figure S6H, Supporting Information). 7) Data download: This module provides open access to all resources generated in this study, including cell type DEG tables, the single cell datasets (IPGCA core, extend, and All), and the three types of eQTL resources.

## 3 Discussion

Significant progress has been made in the integration of atlases in human tissues such as the lung,^[^ ^18^ ^]^ intestine,^[^ ^14^ ^]^ and endometrium,^[^ ^15^ ^]^ yet a comprehensive understanding of farm animals remains lacking. In this study, we present an integrated scRNA‐seq atlas of pig intestinal tract (IPGCA), encompassing multiple segments, stages, and breeds. While individual datasets are often limited by the number of cells and the constraints of their experimental designs, our integrated atlas overcomes these limitations by offering a unified view of cellular diversity and function. We re‐annotated each dataset using a standardized approach, thereby recovering rare cell types that may have been overlooked in individual studies. By combining our atlas with large‐scale genetic studies, we identified cell types that are crucial for understanding complex traits. Additionally, the IPGCA can act as a reference for deconvolution, enabling the parsing of constituent and masked cell type expression discrepancies in bulk RNA‐seq. It also facilitates the mapping of cell type interaction eQTLs and deconvolutional cell type‐specific eQTLs. To further enhance accessibility and utility, we developed an online platform for querying and exploring these datasets. In the future, the IPGCA can also serve as a valuable resource for subsequent tasks such as annotation, querying, mapping, and deconvolution. While data integration and the use of IPGCA have yielded valuable biological insights, several aspects deserve further discussion. For instance, the cell type annotations within IPGCA—especially for immune subsets and functionally distinct epithelial subtypes such as K and L cells (subsets of EECs) will benefit from continuous extension of the datasets. Although multiple batch‐effect correction methods were evaluated, challenges of overcorrection and undercorrection persist. To enhance annotation accuracy, IPGCA must evolve through systematic integration of high‐resolution datasets and multimodal omics data (e.g., spatial transcriptomics, ATAC‐seq), establishing a dynamically updated reference framework.

Although biological factors account for the majority of cell type proportion variance, technical variables introduce systematic biases in specific lineages. Consequently, rigorous bias‐controlled sampling strategies are essential before interpreting compositional differences biologically. We found that stage and breed significantly affect the expression and composition of porcine intestinal cells. Additionally, factors such as weaning status may potentially influence immune and neural cells (Figure 1F). Previous studies have demonstrated that early weaning impacts long‐term systemic immune and neuroendocrine responses to later life challenges in pigs,^[^ ^82^ ^]^ which align with our observations. Crucially, we resolved the longstanding controversy regarding Paneth cell presence in pigs^[^ ^48^ ^]^ through integrated single cell transcriptomics, immunohistochemistry, and immunofluorescence validation across breeds and developmental stages. Immunomodulatory profiling of the weaned piglets captured dynamic B cell lineage remodeling during weaning, including differentiation into *SDC1* ^+^ plasma cells—a transition corroborating findings from Tang et al.^[^ ^56^ ^]^

Pigs are one of the major meat sources and possess numerous economically important gut‐related traits.^[^ ^83^ ^]^ Connecting these traits to single cell data can help elucidate their underlying cellular mechanisms. For instance, we identified associations between porcine complex traits (e.g., loin muscle area) and specific cell types (e.g., tuft and microfold cells). Tuft cells and microfold cells are both important immune surveillance cells in the intestine,^[^ ^84^, ^85^ ^]^ associated with immune stimulation triggered by macromolecules, microbes, and pathogens. Their roles prompt consideration of the interplay between economic traits and immune functions, which has been investigated before.^[^ ^86^, ^87^ ^]^ It's also important to perform functional validation in vitro. Besides, many negative associations remain unexplored, and robust cell‐to‐cell communication pathways underlying single cell or multicellular regulatory mechanisms are yet to be identified.

We performed test of deconvolution on both pseudo‐bulk and real bulk samples. Real bulk and pseudo‐bulk samples exhibit a negative binomial distribution with less overdispersion observed, consistent with human datasets.^[^ ^88^ ^]^ Although the deconvolution performed well in pseudo‐bulk tests, there remains potential for enhancement in real datasets. This may be due to technical discrepancies in sampling and sequencing processes between bulk and corresponding single cell data, leading to relative mismatches in cell proportions and expression levels, as we found that deconvoluting bulk data to the cellular level revealed uneven cell proportions, and cell compositions from different samples of the same tissue also showed a gradient distribution. Therefore, more tests with well‐matched real datasets should be conducted in the future.^[^ ^89^ ^]^

Beyond biological insights, the IPGCA resource holds significant potential for advancing pig breeding programs. Specifically, IPGCA enhances causal mutation discovery by providing cell type‐resolved annotation layers for post‐GWAS functional fine‐mapping. Identified mutations can serve as high‐value genetic markers for marker‐assisted selection or receive prioritized weighting in genomic selection models such as genomic feature best linear unbiased prediction.^[^ ^90^ ^]^ Furthermore, leveraging the demonstrated utility of single cell datasets in refining human polygenic risk scores (PRS) via methods like Yimin et al.,^[^ ^91^ ^]^ we propose adapting this framework to porcine genomics. Integrating IPGCA might improve prediction accuracy for economically critical traits in newborn pigs.

We uncovered high similarities in the molecular profiles of cell types, marker genes, and cellular composition between humans and pigs. Mapping human ulcerative colitis to IPGCA identified associations with CD4^+^ T cells and ILCs (Figure S5E, Supporting Information), consistent with previous reports.^[^ ^92^ ^]^ However, a systematic framework bridging human and porcine gut biology remains underdeveloped, particularly beyond transcriptional correlation analyses. While certain cell types exhibit conserved molecular signatures, the extent of this conservation likely depends on contextual factors, including developmental stage (porcine) versus age (human), breed divergence versus human genetic ancestry, and specific disease states. Elucidating these context‐dependent constraints represents a promising direction for future research. Given pig‐human gut similarities in cell types, these findings support the use of pigs as an in vivo experimental model for humans and their future potential for xenotransplantation applications.

In summary, the results presented herein substantiate the utility and technical efficacy of our integrated single cell RNA sequencing datasets. The reuse of these extensive datasets may facilitate a more profound comprehension of the dissection and application of economically relevant traits in animals, as well as within the realm of animal breeding and experimental animal in vivo models for humans.

## 4 Experimental Section

### Datasets Summary—IPGCA Core Datasets

Given that most studies have focused on the ileum and other small intestinal segments, to supplement the completeness of the datasets, the own sequence data covering five segments (duodenum, jejunum, ileum, cecum, and colon) was generated. For newly generated datasets, total 18 datasets (*n* = 18) included samples from CN pigs (*n* = 3) across a developmental stage range from newborn to adult, as well as from adult wild boar (*n* = 5) and adult Duroc pigs (*n* = 10; 5 lean‐type, D1; 5 obese‐type, D2). In addition, 25 unpublished CN samples from our laboratory to expand and integrate the datasets were incorporated. For public datasets, 7 publicly available single cell sequencing datasets related to pig intestine were collected, comprising a total of 36 intestinal samples, including the GSE162287, GSE193975, GSE163272, GSE174112, GSE175411, GSE196659, and GSE208613 datasets. For the developmental stage classification, the following stage groups were defined: Newborn (0 day), Suckling (0–3 weeks), Weaning (21–35 days or explicitly mentioned in the original paper), Weaner (4–8 weeks), Feeder (8 weeks to 180 days), and Adult (older than 180 days). All of the sample information has been listed in Table S1 (Supporting Information).

### Datasets Summary—IPGCA Extend Datasets

Given that the initial collection of the IPGCA core was limited to data available up to September 2023, CellTypist (v1.7.1) + scANVI (v0.20.3) pipeline to rapidly map subsequent datasets was adopted. The new datasets originate from the following sources: CRA010843, PRJNA907920, GSE232605, GSE293713, GSE233285, CRA018161, and GSE196388. All of the sample information are listed in Table S1 (Supporting Information).

### Datasets Summary—Human scRNA‐Seq Datasets

The human intestinal single cell dataset was collected from the GUT CELL SURVEY (gutcellatlas.org), including a single cell RNA‐seq dataset of 428 K intestinal cells from fetal, pediatric, adult donors, and up to 11 intestinal regions.

### Datasets Summary—Mouse scRNA‐Seq Datasets

The mouse intestinal single cell dataset was collected from Yoon, Seokhyun's figshare dataset^[^ ^79^ ^]^ (https://doi.org/10.6084/m9.figshare.24670038.v1).

### Datasets Summary—Pig Bulk RNA‐Seq Datasets

For RNA‐seq samples, datasets were retrieved comprising 3384 RNA‐seq runs from the NCBI (https://www.ncbi.nlm.nih.gov/sra/) Sequence Read Archive (SRA) by searching the “organism” for “pig”, “tissue” for “gut” or “intestine” and the “strategy” for “RNA seq”. In addition, the own bulk RNA‐seq data, including D1 Duroc and D2 Duroc of five segments (n_duodenum = 96, n_jejunum = 17, n_ileum = 96, n_cecum = 96, n_colon = 18) was generated.

### Datasets Summary—Pig GWAS Datasets

All GWAS summary statistics were from a large‐scale pig metaGWAS project^[^ ^93^ ^]^ and available at PigBiobank^[^ ^68^ ^]^ (https://pigbiobank.farmgtex.org/).

### Datasets Summary—Human GWAS datasets

The GWAS summary statistics file for each trait was downloaded from the UK Biobank database or published studies (https://data.broadinstitute.org/alkesgroup/UKBB).

### Collection of Animal Tissues and Single Cell RNA Sequencing

Chinese indigenous (CN) pigs, neonatal wild boars, and Duroc were employed in the study. The animals were rendered unconscious using CO_2_ anesthesia, followed by bleeding and slaughter in accordance with standard humane protocols, after a fasting period of 12 h. The isolated tissues were rinsed with 1× phosphate buffered saline (PBS) to eliminate contaminants, immediately placed on ice, and embedded using an intestine dissociation kit and subsequently transported to a sterile laboratory environment for further experiments. Tissue samples were placed in gentleMACS C tubes (130‐093‐237; Miltenyi) containing enzyme digestion solution (Hepes, Liberase TM, DNase I in HBSS), dissociated at 37 °C with a gentleMACS Octo Dissociator (130‐095‐235; Miltenyi), and cells were filtered through a 100 µm mesh sieve and collected by centrifugation at 500 g for 5 min at 4 °C. After erythrocyte removal and cell counting, cells were washed twice with Flow Buffer (PBS containing 5% fetal bovine serum and 2 mm EDTA) and resuspended at 1 × 106 cells mL^−1^ in RPMI 1640 medium with 0.04% bovine serum albumin on ice. Single cell RNA sequencing (scRNA‐seq) libraries were prepared using the 10X Genomics Chromium Single Cell 3′ Reagent Kit (version 3) according to the manufacturer's protocol. Sequencing was performed on the Illumina NovaSeq 6000 platform at Novogene to analyze the cellular transcriptome.

### Single Cell RNA Sequencing Data Processing

Public scRNA‐seq datasets and newly generated data were processed using CellRanger^[^ ^94^ ^]^ (v7.0.0), based on the Sus scrofa 11.1 assembly reference genome available from the Ensembl database (gtf version 100). Downstream single cell analysis was conducted using Scanpy^[^ ^95^ ^]^ (v1.8.2), which included quality control (filtering/doublets), normalization, data correction and integration, feature selection, dimensionality reduction, visualization, clustering, and cell type annotation. The scrublet^[^ ^96^ ^]^ module was used to remove doublets, and low‐quality cells were filtered out based on the criteria that the number of genes expressed per cell should be >500 and <7500, with mitochondrial DNA‐derived gene expression <50%. Cell cycle and mitochondrial genes were regressed out using “scanpy.pp.regress_out”. Highly variable genes were selected for dimensionality reduction analysis if they exhibited variability in at least 20 batches. Overall, in IPCGA core dataset, 58 9101 cells passed quality control, in IPGCA extend dataset, 61 6557 cells passed quality control.

### Batch Effect Correction

To address the potential batch effects arising from technical factors in different species and tissues, several commonly used software algorithms were evaluated for batch correction, including: bbknn^[^ ^97^ ^]^ (v1.5.1), scVI^[^ ^26^ ^]^ (v0.20.3), scanorama^[^ ^98^ ^]^ (v1.7.2), harmony^[^ ^25^ ^]^ (v0.0.9), Seurat^[^ ^13^ ^]^ (v4.3), combat^[^ ^99^ ^]^ (v0.3.3), and FastMNN (wrappered in Seurat). For each tissue, these algorithms were applied to correct batch effects and assessed their performance using a combination of internal and external evaluation metrics. Internal metrics included the Silhouette score and Batch entropy mixing score, while external metrics encompassed the adjusted rand index (ARI) and normalized mutual information (NMI). Additionally, the Over‐correction score was calculated to evaluate the balance between correction efficacy and preservation of biological signals. Based on these comprehensive evaluations, the optimal batch correction strategy was selected for each tissue, ensuring robustness in downstream analyses.

### Cell Type Annotation

To ensure accurate and precise atlas annotation, cells at three hierarchical levels were annotated: lineage, cell type, and sub‐cell type. A combination of automatic and manual annotation methods was employed. For manual annotation, the original publications were referred and utilized the CellMarker^[^ ^100^ ^]^ database (http://117.50.127.228/CellMarker/). Automatic annotation was based on public annotated datasets to learn global expression patterns and predict possible cell types by CellTpyist.^[^ ^25^ ^]^ The annotated data were then used to train models that could predict cell types in further datasets.

At last, cells were classified into eight major lineages at the first level using classical markers^[^ ^30^, ^31^, ^36^, ^40^, ^101^, ^102^, ^103^, ^104^, ^105^, ^106^ ^]^ to identify the epithelial, endothelial, T/ILC, B, myeloid, mesenchymal, neuron, and red blood lineages. At the second level, each lineage was divided into major cell types based on specifically expressed markers. At the third level, these cell types were further subclustered to identify heterogeneity within each type. Detailed cell type markers are listed below.

### Cell Type Annotation—Epithelial Lineage

The epithelial lineage (*EPCAM^+^*) can be further classified into stem (*LGR5+*, *OLFM4^high^*, and *HMGB2^high^*), TA (*TOP2A^+^, PCNA^+^*, and *DUT^high^*), immature Enterocytes (stem and enterocytes markers expressed concurrently), Enterocytes (*FABP2^+^, ANPEP^high^*, and *RBP2^+^*), colonocytes (*SLC26A2^+^* and *CA2^high^*), BEST4 enterocytes (*BEST4^+^* and *OTOP2^+^*), EECs (*CHGA^+^, CHGB^+^*, and *NEUROD1^+^*), goblet cells (*CLCA1* ^+^, *SPDEF* ^high^, *TFF3* ^high^, and *REG4* ^high^), microfold cells (*GP2^+^*), Paneth cells (*PIGR^high^, CPSF6^high^*, and *MMP7^+^*), tuft cells (*POU2F3^+^* and *IRAG2^+^*).

### Cell Type Annotation—Endothelial Lineage

The endothelial lineage (*PECAM1^+^*) can be further divided into venous (*ACKR1^+^*), arterial (*GJA4^+^, HEY1^+^, HEY2^+^*, and *EFNB2^high^*), and lymphatic endothelium (*PROX1^+^, LYVE1^+^*, and *CCL21^+^*) subtypes.

### Cell Type Annotation—T/ILC Lymphoid Lineage

The T/ILC lymphoid lineage (*CD3E^+^* and *CD3G^+^*) can be subdivided into CD4+ T cells (*CD4^+^* and *CD28^+^*), CD8+ T cells (*CD8A^high^* and *CD8B^+^*), MEF2C+ T cells (*MEF2C^+^* and *FGR^high^*), cytotoxic T cells (*TOX^high^, GZMA^high^, GZMH^high^*, and *FCER1G^high^*), ILC (*KLRB1^high^, RORC^+^, KIT^high^, IL23R^+^*, and *CXCL2^+^*), NK cells (*NKG7^high^, PRF1^high^, GNLY^high^*, and *KLRB1^high^*).

### Cell Type Annotation—B lymphoid Lineage

The B lymphoid lineage (*CD19^+^* and *CD79^+^*) can be further categorized into activated B cells (*AICDA^high^, BCL6^high^*, and *CD86^high^*), cycling B cells (*TOP2A^+^, STMN1^+^, BIRC5^+^*, and *PCLAF^+^*), resting B cells (*KLF2^+^, CCR7^+^*, and *SELL^+^*), plasma cells (*JCHAIN^high^, XBP1^+^, IRF4^+^*, and *PRDM1^+^*).

### Cell Type Annotation—Myeloid Lineage

The myeloid lineage (*CD163* ^+^ and *CD68* ^+^) can be further classified into macrophage (*CD68* ^+^, *APOE* ^+^, *C1QA* ^+^, *CQ1B* ^+^, and *C1QC* ^+^), mast (*LTC4S* ^+^ and *CCN3* ^+^), monocyte (*CEBPB* ^high^, *S100A12* ^+^, and *ENSSSCG00000029414* ^+^), dendritic (*GSN* ^high^, *CEBPE* ^+^, *XCR1* ^+^, and *SNX22* ^+^), plasmacytoid DCs (*VPREB1* ^+^, *IRF7* ^high^, and *MZB1* ^high^).

### Cell Type Annotation—Mesenchymal Lineage

The mesenchymal lineage (*COL3A1^+^* and *COL1A2^+^*) can be further divided into myofibroblasts (*ACTA2^high^* and *TAGLN^high^*), fibroblast (*DCN^high^, BMP5^high^*, and *C7^high^*), smooth muscle (*CNN1^+^, ACTA2^high^, MYH11^high^, MYOCD^+^*, and *DES^+^*), myenteric glia (*CD9^high^* and *GFAP^+^*), pericyte (*NOTCH3^+^, MCAM^+^, RGS5^high^, KCNJ8^+^*, and *ABCC9^+^*), C4A+ stromal (*CLU^high^, C3^high^*, and *C4A^high^*).

### Cell Type Annotation—Neuron and blood

The neuronal lineage (*SOX10^+^*) can be further subdivided into Glial (*GFAP^+^*), neuron (*GAP43^+^*). And the red blood cells were identified by *HBB*.

All of the annotation details have been listed in Table S2 (Supporting Information).

### Cell Type Label Transfer and Extension of the IPGCA Core

To expand our IPGCA dataset, a CellTypist (v1.7.1) + scANVI (v0.20.3) pipeline was employed to map aforementioned single cell datasets after September 2023 and integrate them with the IPGCA core. CellTypist was first used to train an automatic annotation pkl model for each cell lineage in the IPGCA core. For new datasets, the same preprocessing standards were applied as the IPGCA core, including quality control, data normalization, batch correction, and dimensionality reduction and clustering. After delineating lineages using canonical lineage marker genes (Table S2, Supporting Information), each lineage was subsetted for further dimensionality reduction and clustering, and then performed label transfer using the pre‐trained pkl model corresponding to each lineage. Clusters were selected with Leiden algorithm (resolution = 1) for majority voting and retained only cell labels with a confidence score greater than 0.8 to ensure annotation accuracy. The newly generated dataset is termed IPGCA extend. Finally, scANVI integration was performed using IPGCA core as reference and IPGCA Extend as query, with cell type‐guided batch correction producing the consolidated IPGCA All resource.

### Variance Between Individuals Explained by Covariates

The variance explained by each covariate for each cell type to quantify the contribution of technical and biological covariates was computed. The impact of covariates at the cellular lineage level by performing principal component regression on every covariate was investigated, which yielded the fraction of latent component variance explained per covariate. Samples lacking values for a given covariate (e.g., where the sex was not recorded for some samples) were excluded from the regression. Categorical covariates were converted to dummy variables.

### Differential Analysis and Functional Enrichment Analysis

Differential expression was assessed with Scanpy's “sc.tl.rank_genes_groups” using the “Wilcoxon” test; *p* values were “Benjamini–Hochberg” adjusted and filtered at FDR < 0.01. These DEGs were subsequently subjected to enrichment analysis. GO and KEGG enrichment analyses were conducted by clusterProfiler^[^ ^107^ ^]^ (v 4.14.4). The Benjamini–Hochberg (BH) procedure was applied for multiple testing corrections, with only GO terms having an adjusted *p* values below 0.05 being retained.

### Immunohistochemistry and Immunofluorescence

After collecting the tissue samples, these were gently rinsed with PBS to remove impurities and immediately placed into PBS‐based (G0002; Servicebio) tissue fixative. Subsequently, the fixed tissues were processed for paraffin embedding. The immunohistochemical staining protocol begins with de‐paraffinization, where sections were de‐waxed through two incubations in xylene (10023418; Sinopharm) (15 min each), followed by 85% and 75% ethanol (5 min each), and finally rinsed in distilled water. For antigen retrieval (G1202; Servicebio), slides were placed in citrate buffer (pH 6.0) and heated in a microwave for antigen retrieval, then cooled in PBS (pH 7.4) with gentle shaking. Endogenous peroxidase activity is quenched by incubating in 3% hydrogen peroxide for 25 min, followed by rinsing in PBS. After draining excess liquid, sections were encircled with a PAP pen and incubated with 3% BSA for 30 min at room temperature, using rabbit serum (G1209; Servicebio). Once the blocking solution was discarded, primary antibodies diluted in PBS were applied and incubated at 4 °C overnight in a humid chamber. Following rinses in PBS, sections were incubated with HRP‐conjugated (GB23303; Servicebio) secondary antibodies for 50 min at room temperature. After rinsing in PBS, 3,3′‐diaminobenzidine (DAB) substrate (G1212; Servicebio) was applied for visualization, with color development monitored microscopically and terminated by a water rinse. Nuclei were then counterstained with hematoxylene (G1004; Servicebio), differentiated, and blued in Scott's tap water, followed by washing. Finally, slides were dehydrated through graded ethanol and xylene, air‐dried, and mounted with neutral resin. The primary antibodies used include anti‐LYZ (1:5000; GB11345; Servicebio) and anti‐MMP7 (1:100; AB232737; Abcam).

The immunofluorescence protocol begins with samples being submerged in 4% paraformaldehyde (PFA) (G1128; Servicebio) overnight at 4 °C, followed by embedding in paraffin and sectioning into 5 µm thick slices. After deparaffinization and antigen retrieval using a commercial kit (G1202; Servicebio), the sections were blocked with 3% bovine serum albumin (BSA) (GC305010; Servicebio) for 30 min at room temperature. Subsequently, the sections were incubated with primary antibodies overnight at 4 °C. The samples were then washed with PBS and incubated with secondary antibodies in the dark for 1 h at room temperature. After washing, the slides were stained with 4′,6‐diamidino‐2‐phenylindole (DAPI) (G1012; Servicebio) and mounted with a mounting solution (G1221; Servicebio). The primary antibodies used include anti‐EPCAM (1:5000; GB11274; Servicebio), anti‐LYZ (1:5000; GB11345; Servicebio), anti‐MMP7 (1:100; AB232737; Abcam), anti‐MEF2C (1:500; GB11692; Servicebio), and anti‐CD3E (1:500; GB150141; Servicebio).

### Selection Pressure Analysis

The genotype dataset was from Pig Haplotype Reference Panel (PHARP).^[^ ^52^ ^]^ The CN pigs (*n* = 228) and wild boars (*n* = 125) were extracted by PLINK (v1.90)^[^ ^108^ ^]^ and parameter “–hwe” were set to 1e‐6, “–mac” to 6, and “–maf ” to 0.05. Then eigenGWAS^[^ ^50^ ^]^ and *F*_ST_^[^ ^51^ ^]^ analysis were conducted by gear2. The xpclr^[^ ^49^ ^]^ (v1.1.2) software was used to conduct XP‐CLR analysis with 50 kb windows and 20 kb steps. Then, genomic regions were deemed to be under selection only when all three metrics simultaneously ranked within the top 1%. The candidate regions were annotated using GALLO^[^ ^109^ ^]^ (v1.5). Enrichment analysis of putative selective‐sweep regions within Paneth cell DEGs was performed via a one‐sided hypergeometric test. The background reference set comprised all high‐confidence DEGs across cell types (*n* = 3258), including 100 Paneth cell‐specific DEGs. Of 602 genes in selective‐sweep regions, 26 overlapped with the Paneth cell DEG set. This analysis was conducted in SciPy (v1.11). Unless otherwise specified, all *p* values are two‐tailed, with statistical significance defined as *p* < 0.05.

### RNA Velocity and Pseudo Time Analyses

For each lineage with differentiated cell types, cell trajectory analysis of gene RNA velocity was conducted. Initially, the sorted genome bam file was processed using velocyto.py to obtain a loom file to get splice and unspliced information. Subsequent analysis was performed using the scVelo^[^ ^55^ ^]^ package. After merging the preprocessed adata object with the loom file object, filtering and normalization were applied using “scv.pp.Filter_and_normalize”, followed by “scv.pp.moments” function. After that, gene‐specific velocities were obtained using “scv.tl.velocity (mode = “stochastic”)”. Additionally, “scv.tl.velocity_graph” was used to fit the ratio between unspliced and spliced mRNA abundances. Visualization of RNA velocities was achieved using either “scv.pl.velocity_graph” or “scv.pl.velocity_embedding_grid”. This allowed us to validate subpopulation identification based on the inferred temporal relationships. CellRank2 was applied to delineate the B‐lineage trajectory and quantify fate probabilities. After extracting B lineage cells from IPGCA, the “VelocityKernel” was used to predict terminal states, compute fate probabilities, and identify lineage‐driver genes along the pseudotime.

### Single Cell Regulatory Network Analysis

hdWGCNA^[^ ^57^ ^]^ is a tool that offers various functions for network inference, gene module identification, functional gene enrichment analysis, network reproducibility statistical testing, and data visualization. Uncovering gene module information is crucial for investigating the roles and significance of key genes, signaling pathways, and gene‐gene regulatory mechanisms. In this analysis, “SetupForWGCNA” was employed to construct a gene co‐expression network based on genes expressed in at least 5% of cells within Seurat objects. Sample clustering to exclude outliers was initially performed. The resulting WGCNA tree had a minimum module size of 50 genes, integrating similar genes into distinct modules. The “plot_dendrogram” function was used to visualize the co‐expression modules generated by network analysis. Each leaf on the tree represents a gene, with colors at the bottom indicating assignment to specific co‐expression modules. Finally, the “GetMEs” function was used to visualize the expression levels (module eigengenes, ME) of each module.

### Bulk RNA‐Seq Processing

A stringent and uniform pipeline identical to PigGTEx to filter and analyze all the data was applied. First, adapters and low‐quality reads using Trimmomatic^[^ ^110^ ^]^ (v.0.39) were removed. Clean reads were then mapped to the Sscrofa11.1 pig reference genome^[^ ^111^ ^]^ (gtf verison 108) using HISAT2^[^ ^112^ ^]^ (v2.2.1). The 1425 samples were retained with more than 500000 clean reads and uniquely mapping rates of at least 60% for subsequent analysis. Raw counts were obtained for 35671 Ensembl (Sscrofa11.1 v108) genes using featureCounts^[^ ^113^ ^]^ (v2.0.1) and calculated the expression levels of the transcripts in transcripts per million (TPM) and fragments per kilobase of transcript per million mapped reads (FPKM) using StringTie^[^ ^114^ ^]^ (v2.1.7). To visualize gene expression variability across samples, t‐distributed stochastic neighbor embedding (t‐SNE) with a distance metric of 1−r was applied, where r represents Pearson's correlation coefficient of gene expression. This step also helped rectify potentially mislabeled samples using the Leiden clustering method. To address potential discrepancies in the specific locations and labels of intestinal samples, a clustering approach was employed to identify and exclude outlier samples. Samples from the IPEC‐J2 cell line and colon neurons were identified as outliers and subsequently removed from the intestinal cis‐QTL analysis, which was then performed separately for these distinct samples.

### Cellular Deconvolution Benchmark Analysis

The application of human transcriptome data for deconvolution has been widely investigated; however, this study evaluates the performance of various deconvolution tools using a porcine dataset. Several tools were tested, including MuSiC2,^[^ ^115^ ^]^ BayesPrism,^[^ ^23^ ^]^ TAPE,^[^ ^116^ ^]^ and SCDC.^[^ ^117^ ^]^ To assess their performance, a single cell dataset was first utilized to simulate bulk data using decoupler^[^ ^118^ ^]^ (v1.6.0) with the mode set to “sum”. The cell composition proportions derived from the single cell data served as the ground truth. The correlation between the cell proportions estimated by each software and the ground truth using Pearson's correlation coefficient was calculated. Additionally, metrics were employed from the CATD^[^ ^88^ ^]^ pipeline to evaluate the effectiveness of the deconvolution process.

The potential impact of discrepancies between single cell and bulk RNA sequencing on the accuracy of deconvolution analysis was also investigated. A comparative assessment of gene expression deviations was conducted by using single cell data as a reference and comparing it with corresponding bulk data in terms of mean absolute deviation (mAD). This comparison was visualized through scatter plots of the mean and variance of expression data. Ultimately, predicted cell composition ratios for all processed bulk RNA‐seq using BayesPrism were obtained.^[^ ^23^ ^]^

### Genotyping and Imputation From RNA‐Seq Samples

The GATK^[^ ^119^ ^]^ (v4.0.8.1) to call SNPs at known loci in the dbSNP database (build 150), according to the recommended settings of the GATK best practice guidelines was employed. Low‐quality SNPs were filtered out using the filtering option: FS > 30.0 & QD < 2.0 & DP < 6.0. After that, the filtered SNPs was imputed on autosomes using Beagle^[^ ^120^ ^]^ (v5.1) based on haplotypes from the PGRP.^[^ ^11^ ^]^ Finally, variants were filtered out with MAF < 0.05, MAC < 6 and model‐based imputation accuracy (DR2) < 0.8, resulting in 9086656 SNPs for the molQTL mapping.

### Detection of Duplicated and Mis‐labelled RNA‐Seq Samples Within Each Tissue

The identity‐by‐state (IBS) distance among samples from LD‐independent SNPs to remove samples from the same individuals within each tissue using PLINK^[^ ^108^ ^]^ (v1.90) was calculated. “–genome–genome‐full” parameters were used to get the ***PI_HA***

(1) $$PI\_HAT=\frac{\left(IBS2+0.5\times IBS1\right)}{\left(IBS0+IBS1+IBS2\right)}$$

where ***IBS*0** is the number of non‐missing variants with ***IBS*** = 0 (two different alleles), ***IBS*1** is the number of non‐missing variants with ***IBS*** = 1 (one shared allele), and ***IBS*2** is the number of non‐missing variants with ***IBS*** = 2 (two shared alleles). When the ***PI_HAT*** value between two samples exceeds 0.9, they are considered duplicate samples. After merging replicates samples, resulting in 1464 distinct RNA‐seq samples.

### Molecular eQTL Mapping—Tissue‐level eQTL Mapping

Sample expression was normalized within each tissue using the TMM method from the edgeR^[^ ^121^ ^]^ package. To satisfy the linear model assumptions of homoscedasticity and Gaussian residuals, an inverse normal transformation was applied to the TMM‐normalized matrix. After that, cis‐eQTL mapping was performed using the linear regression model implemented in tensorQTL^[^ ^122^ ^]^ (v1.0.3), incorporating estimated covariates. Within each tissue, genes were filtered out with a TPM value of less than 0.1 or raw read counts of less than 6 reads in more than 80% of samples. Subsequently, a two‐tiered multiple testing correction was applied based on a permutation approach, as implemented in tensorQTL.

### Molecular eQTL Mapping—Cell Type Interaction eQTL Mapping

To determine if cis‐eQTLs exert a significant influence on gene expression within specific cell types, this study conducted cell type‐specific cis‐eQTL (ieQTL) mapping based on the following linear regression model implemented in tensorQTL:

(2) y=A+g+i+g×i+e

where ***y*** represents the vector of gene expression values (i.e., inverse normal‐transformed TMM), ***g*** denotes the genotype dosage (0, 1, or 2) for the tested SNP from RNA samples, ***i*** is the enrichment score (i.e., an inverse‐normalized value representing cell type proportions predicted from scRNA‐seq data using BayesPrism^[^ ^23^ ^]^), ***g* × *i*** represents the interaction term between genotype dosage and enrichment score, and ***A*** signifies the covariates (e.g., genotype principal components and PEER factors) and ***e*** is the residual error.

### Molecular eQTL Mapping—Deconvolutional Cell Type cis‐eQTL Mapping

The “adata.X” was extracted from the IPGCA atlas as a counts matrix and obtained the bulk RNA‐seq expression from aforementioned process. Then, “plot.scRNA.outlier” and “plot.bulk.outlier” were employed to filter out outlier genes (with parameters outlier.cut = 0.01 and outlier.fraction = 0.1) and retained only protein‐coding genes for subsequent deconvolution. Thereafter, “get.fraction” was utilized to extract the posterior mean of cell type fractions and “get.exp” to obtain the posterior mean of the cell type‐specific gene expression count matrix Z. With the deconvoluted cell expression matrix, the gene expression variance was visualized among samples using UMAP. After filtering out outliers within each cell type and categorizing by different tissues and cell types, the aforementioned cis‐eQTL mapping process to perform decon‐eQTL mapping was proceeded.

### Estimation of Effect Sizes of cis‐eQTL

To estimate the effect size of significant cis‐eQTLs, the log allelic fold change (slope_aFC) was computed using the aFC.py tool from the aFC^[^ ^123^ ^]^ (https://github.com/secastel/aFC) for the top variant of each independent cis‐eQTL. The normalized cis‐eQTL effect sizes to the annotation category of the top variant to investigate functional consequences were compared.

### Co‐Localization

Co‐localization analyses was performed between GWAS and eQTL signals for genes harboring at least ten variants using the “coloc.abf” function from the COLOC^[^ ^124^ ^]^ R package (v.5.2.3), employing default priors and beta coefficients derived from both GWAS and eQTL analyses. Initially, a gene‐level co‐localization analysis was conducted between the PigBiobank GWAS summary statistics and all cell type cis‐eQTLs, identifying significant co‐localization events with a posterior probability (PPH4) exceeding 0.9. Subsequently, SNP‐level results and defined events were extracted as significant if both the GWAS summary *p* values and cis‐eQTL *p* values were below the threshold of 5 × 10^−8^. These significant events were then tallied for further analysis. For independent variants of metaGWAS, a method was applied as PigBiobank paper^[^ ^93^ ^]^ did (https://github.com/SCAU‐AnimalGenetics/Pig‐metaGWAS). Then the same co‐localization analyses were performed between three types of eQTLs and these independent loci.

### Complex Traits Enrichment—scDRS Enrichment

GWAS summary statistics were utilized to identify potential trait‐associated genes using MAGMA^[^ ^69^ ^]^ (v 1.10). Most of used GWAS summary statistics were based on the meta‐GWAS across different breeds (Table S5, Supporting Information), and the number of SNPs for these traits ranged from 8 711 178 to 2 7288 580. Each potential disease gene was assigned a weight based on its GWAS MAGMA z‐score and inversely adjusted by its gene‐specific technical noise level in scRNA‐seq. Next, trait‐relevant scores (Disease risk score) and control scores were calculated using the transcriptional profiles of individual cells via scDRS,^[^ ^20^ ^]^ and then computed the corresponding *p* values from these scores. These scores were used in downstream analyses to establish associations between cell types and traits, as well as to examine co‐expression between prioritized disease/trait‐relevant genes and genes implicated by GWASs. For bulk RNA‐seq enrichment using scDRS, counts matrix to build “anndata” object, and performed single cell quality control parameters and subsequent enrichment analysis was first used.

### Complex Traits Enrichment—Heritability Enrichment Analysis

Linkage disequilibrium score regression analysis using LDSC‐seg to perform heritability enrichment analysis was employed. Pig genes were converted to their human homologs using Biomart, and only one‐to‐one orthologs were retained based on gene ID. A baseline was created for 16 cell types and partitioned the heritability based on the second level of annotations. Heritability enrichment was quantified by the ratio of the trait heritability attributed to SNPs within a specific annotation to the overall heritability proportion of all SNPs within the same annotation. The summary statistics file for each trait was downloaded from the UK Biobank database or published studies). Subsequently, these traits were categorized and performed cell type‐specific enrichment. Finally, the coefficient *p* value was reported as a measure of the association of each cell type with the traits.

### Web Construction

A Streamlit was utilized to build the frontend interface of scGutDB and deployed it on an Ubuntu v20.04 server using Apache v2.4.29. The following data resources were provided in the website: Cell type–specific differentially expressed genes, the IPGCA core and IPGCA extend datasets, three types of eQTL, a well‐trained annotation pkl file. To facilitate web‐based queries, the IPGCA core dataset was downsampled to a level of 50 000 cells. The backend plotting scripts were written using the core plotting modules of Scanpy. All of the back‐end processing code is available on GitHub repository (https://github.com/curryfly5/scGutDB).

### Statistical Analysis

Statistical analyses were performed in Python (v3.11) using SciPy (v1.11) and statsmodels (v0.14). Summary statistics are presented as mean ± standard deviation (SD). Comparisons among three or more groups employed one‐way ANOVA; when significant main effects were detected (*p* value < 0.05), Tukey's HSD post‐hoc test with Benjamini–Hochberg correction (FDR < 0.05) was applied. Two independent groups were compared with an unpaired two‐tailed Student's *t*‐test, while paired data were analyzed with paired *t*‐tests. Repeated‐measures designs were analyzed by repeated‐measures ANOVA.

### Ethics Approval

All procedures involving animals adhered to the guidelines for the care of local animal welfare regulations. This study was approved by the Zhejiang University Laboratory Animal Welfare Ethics Review Committee. Our project number is ZJU20230047, which can be found at the Zhejiang University Laboratory Animal Center website (https://www.lac.zju.edu.cn/).

### Code Availability

The IPGCA pipeline for processing the IPGCA datasets (**Experimental Section**), is available at https://github.com/curryfly5/IPGCA_reproducibility. All further codes used for the auto annotation and GWAS enrichment analysis is available at scGutDB (http://alphaindex.zju.edu.cn/scgut/). The codes for the construction of scGutDB can be found at https://github.com/curryfly5/scGutDB.

## Conflict of Interest

The authors declare no conflict of interest.

## Supporting information



Supporting Information

Supporting Information

## Data Availability

The public scRNA‐seq data used in this study were obtained from NCBI, CNGB, GEO and GSA database, including the GSE162287, GSE193975, GSE163272, GSE174112, GSE175411, GSE196659, GSE208613, CRA010843, PRJNA907920, GSE232605, GSE293713, GSE233285, CRA018161 and GSE196388 datasets. Our own generated scRNA‐seq data are available from the corresponding author upon reasonable request. The whole expression matrix and the processed IPGCA resource could be downloaded from scGutDB (http://alphaindex.zju.edu.cn/scgut/).
