# Supplementary material for: IPGCA: A Comprehensive Single Cell Atlas of 1 074 127 Porcine Intestinal Cells Revealing Cellular Dynamics, Genetic Regulation, and Cross‐Species Conservation
Source: Adv Sci (Weinh). 2025 Oct 5;12(47):e07882. doi: 10.1002/advs.202507882 (PMC12713100; doi:10.1002/advs.202507882)
Supplement: Supplementary file 1 — Supporting Information [file ADVS-12-e07882-s001.docx]

**Supplementary figures and tables**


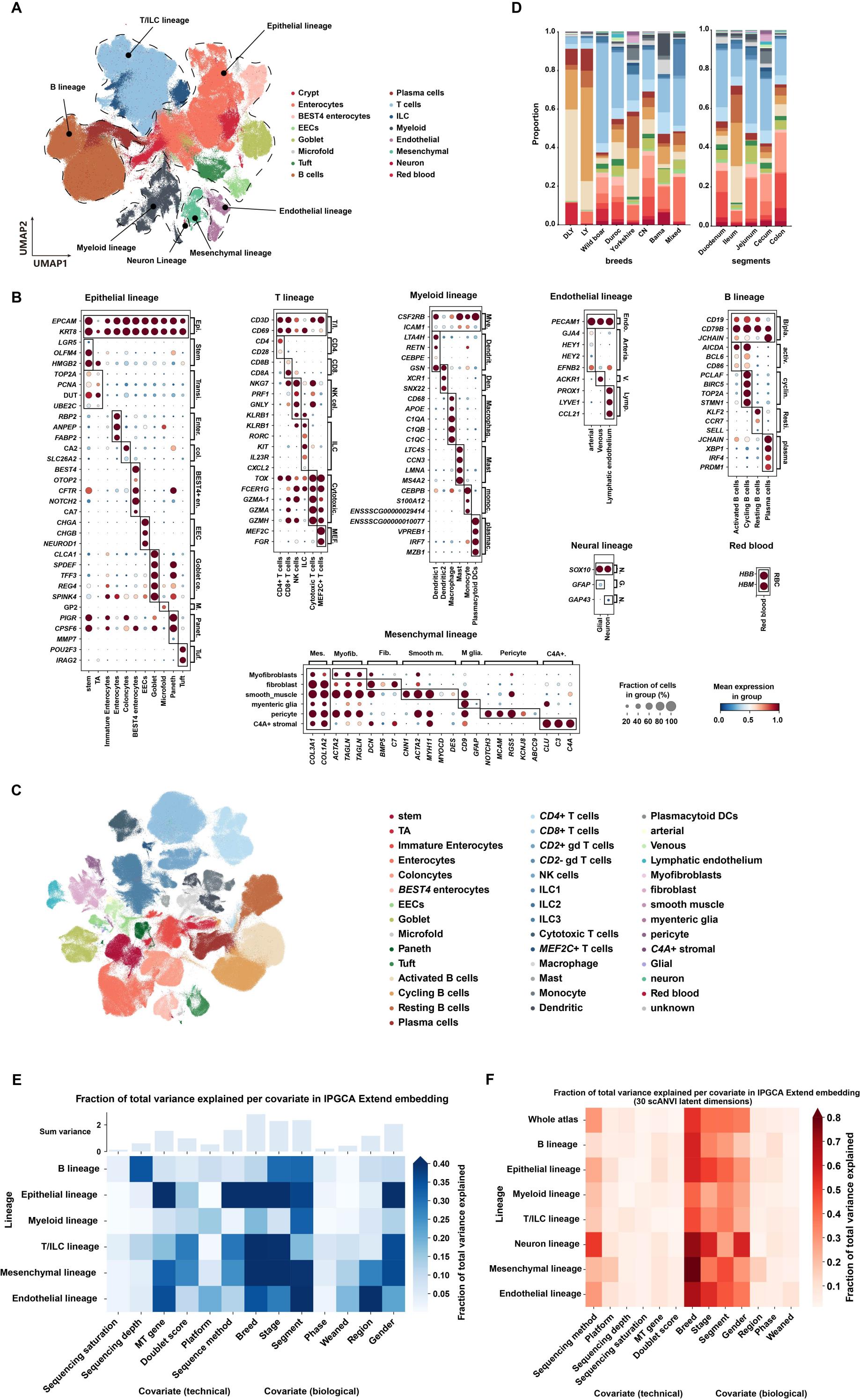


**Figure S1 Cell type annotation maker genes of cell types and benchmarking of different tissues for 7 batch correction methods.**

**A)** Level2 annotation of IPGCA core. **B)** Dotplot for expression of marker genes of cell types and states in each cell lineage in IPGCA core. **C)** The UMAP plot of IPGCA All dataset, comprising both the IPGCA core and IPGCA extend. **D)** Distribution of IPGCA cell types along with different breeds and segments. **E)** The fraction of total inter-sample proportion variance in the IPGCA extend correlating with specific covariates. Covariates are categorized into technical and biological. **F)** The fraction of total inter-sample expression variance in the IPGCA extend with these covariates.


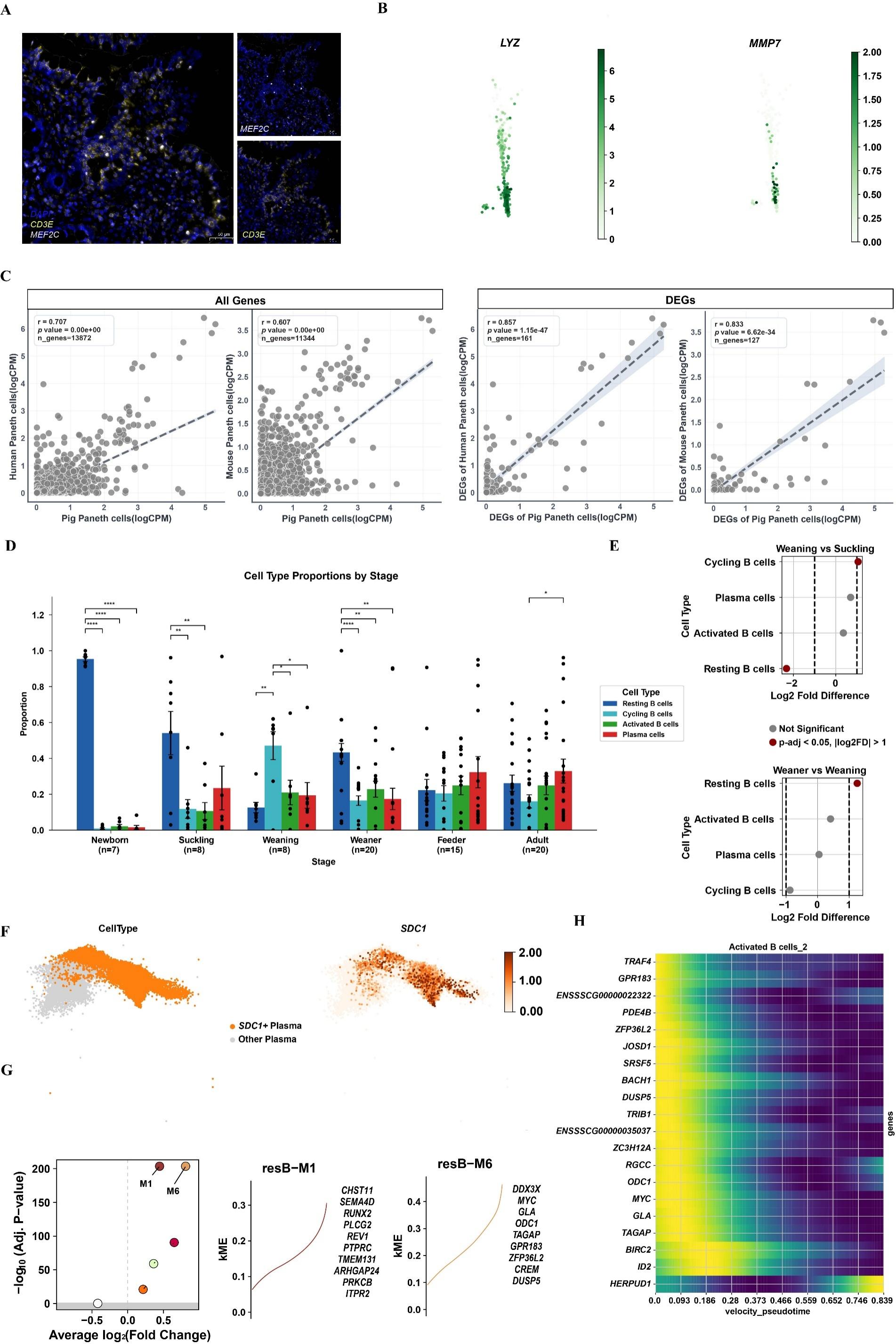


**Figure S2. Proportion discrepancy and gene modules of B cell lineage.**

**A)** Immunofluorescence stained images for *MEF2C^+^* T cell marker, DAPI (Blue), *MEF2C* (White), *CD3E* (Yellow), scale bars = 50 µm. **B)** The feature map of *LYZ* and *MMP7* expression pattern in Paneth cell. **C)** The correlation of all one-to-one mapped homologous genes expression between Paneth cell of pig versus human and pig versus mouse (left); The correlation of shared differential expressed genes expression between Paneth cell in the same comparisons (right). **D)** The boxplot shows the proportions of B lineage cell types across six developmental stages, stratified by scRNA-seq dataset. **E)** The cell proportion analysis between weaning verse suckling piglet (top) and weaner (bottom) verse weaning from 1,000 permutations using scProportionTest. **F)** The annotation of the *SDC1*^+^ Plasma cells, the left figure shows the subset cluster of plasma and right figure shows the feature map of the marker *SDC1*^+^ (*CD138*). **G)** The left figure shows the hd-WGCNA differentially expressed module of resting B cells before and after weaning. The right figure shows the hd-WGCNA differential expressed module of resting B cells before and after weaning and the kME (eigengene-based connectivity) of two module genes. **H)** The Heatmap of the expression along with velocity pseudotime of core genes from the M6 module in “activated B cells_2” trajectory.


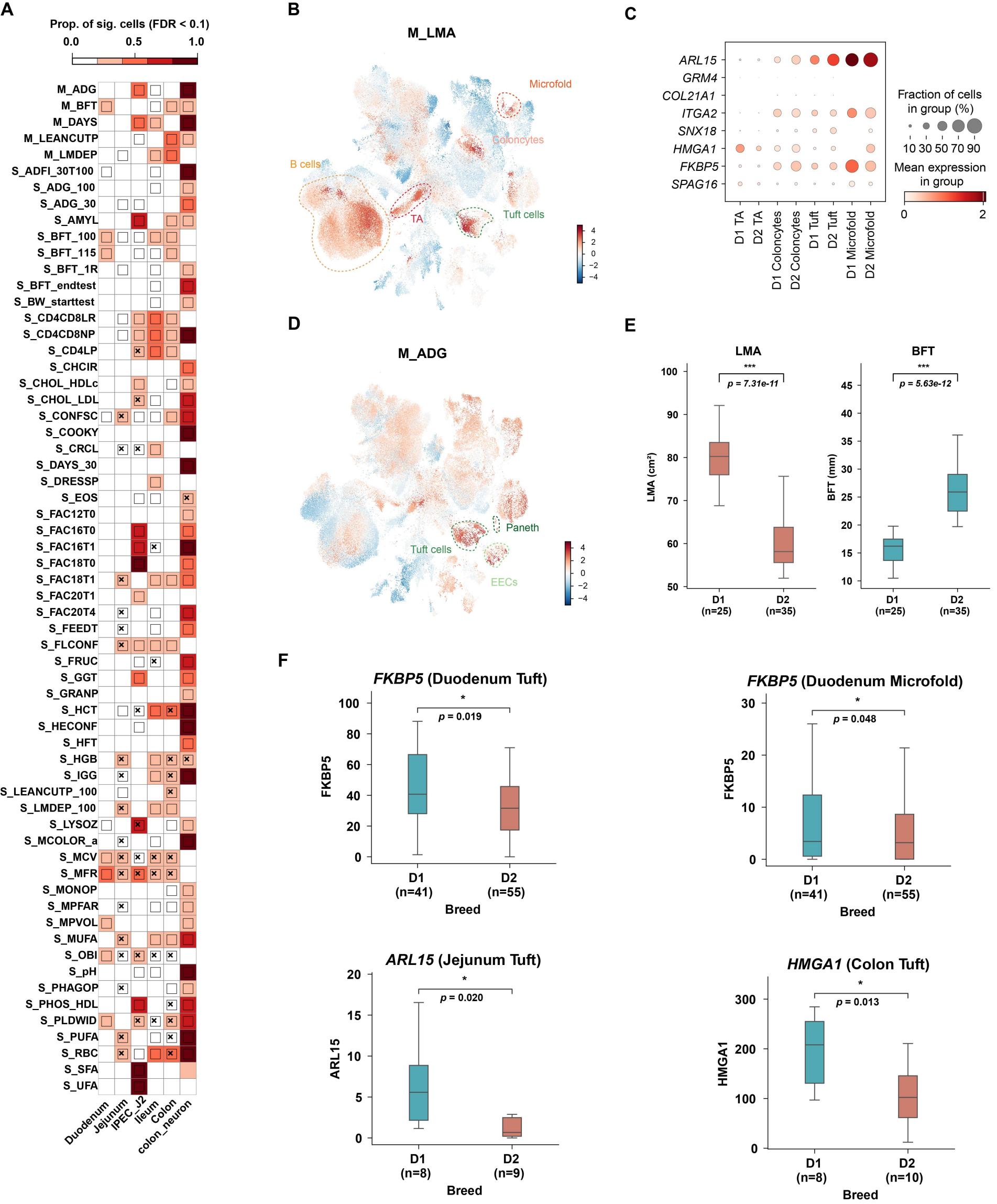


**Figure S3. scDRS score of some economical traits and genes differentially expression in two Duroc lines.**

**A)** Enrichment results of scDRS for 6 tissues/cell lines and complex traits which shows at least one association from PigBiobank. **B)** UMAP of loin muscle area scDRS trait score, positive score is with red shade and negative with blue. **C)** Dotplot of top M_LMA trait-associated MAGMA genes’ expression patterns in associated cell types. **D)** scDRS score of M_ADG, positive score is with red shade and negative with blue. The cell types which are significant in scDRS analysis are circled. **E)** The box plot of the difference in loin muscle area (LMA) and backfat thickness (BFT) between the two Duroc breeds (D1 and D2). **F)** The box plots displaying the differential expression of MAGMA top genes from L_LMA in deconvoluted bulk RNA-seq data for tuft and microfold cell types, the number of the samples listed below (n_phenotype of LMA = 60, n_duodenum = 96, n_jejunum = 17, n_colon = 18).


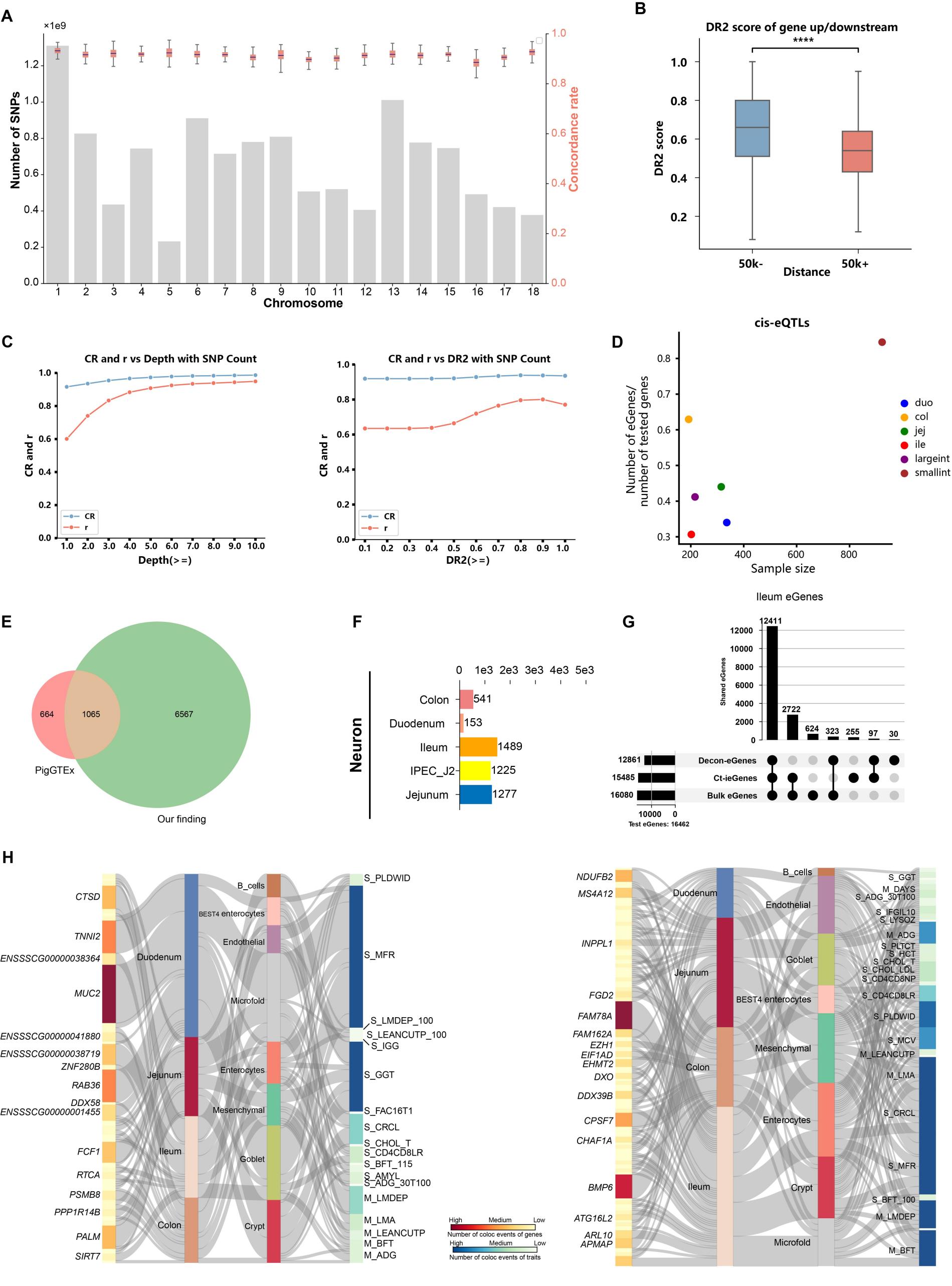


**Figure S4. Imputation accuracy and cis-eQTL exploration.**

**A)** Number of imputed SNPs (gray bars) from 300 FAANG project samples across 18 pig chromosomes after quality control (DR2 ≥ 0.9, MAF ≥ 0.05, MAC≥ 6) and the imputation accuracy (concordance rate). **B)** Distribution of DR2 values for imputed genotypes within the 50-kb upstream and downstream regions of the transcription start site (TSS). **C)** The left figure shows concordance rate (CR) and Pearson's correlation coefficient (r) of observed genotypes increase with sequencing depth. The right figure shows the CR and r of imputed genotypes increase with DR² value. **D)** Pearson’s correlation coefficient (r) between the proportion of detectable eGenes and sample size in bulk eQTL mapping. **E)** Intersection of eGenes identified in PigGTEx and our study. **F)** Number of cell type-specific ieQTLs discovered in neurons at FDR < 0.05. **G)** The barplot shows the count of eGenes that shared among eQTL types (bulk eQTL, ct-ieQTL, and decon-eQTL). Each vertical bar represents a combination, and the horizontal bar (left axis) indicates the corresponding number of eGenes. **H)** The left figure shows co-localization events for decon-eGenes in SNP significance (PPH4 > 0.9, and *p* value of GWAS loci and eQTL in corresponding event <5×10^-8^. The thickness of each flow indicates the number of events); the right figure shows co-localization events for decon-eGenes in eGene significance (PPH4 > 0.9).


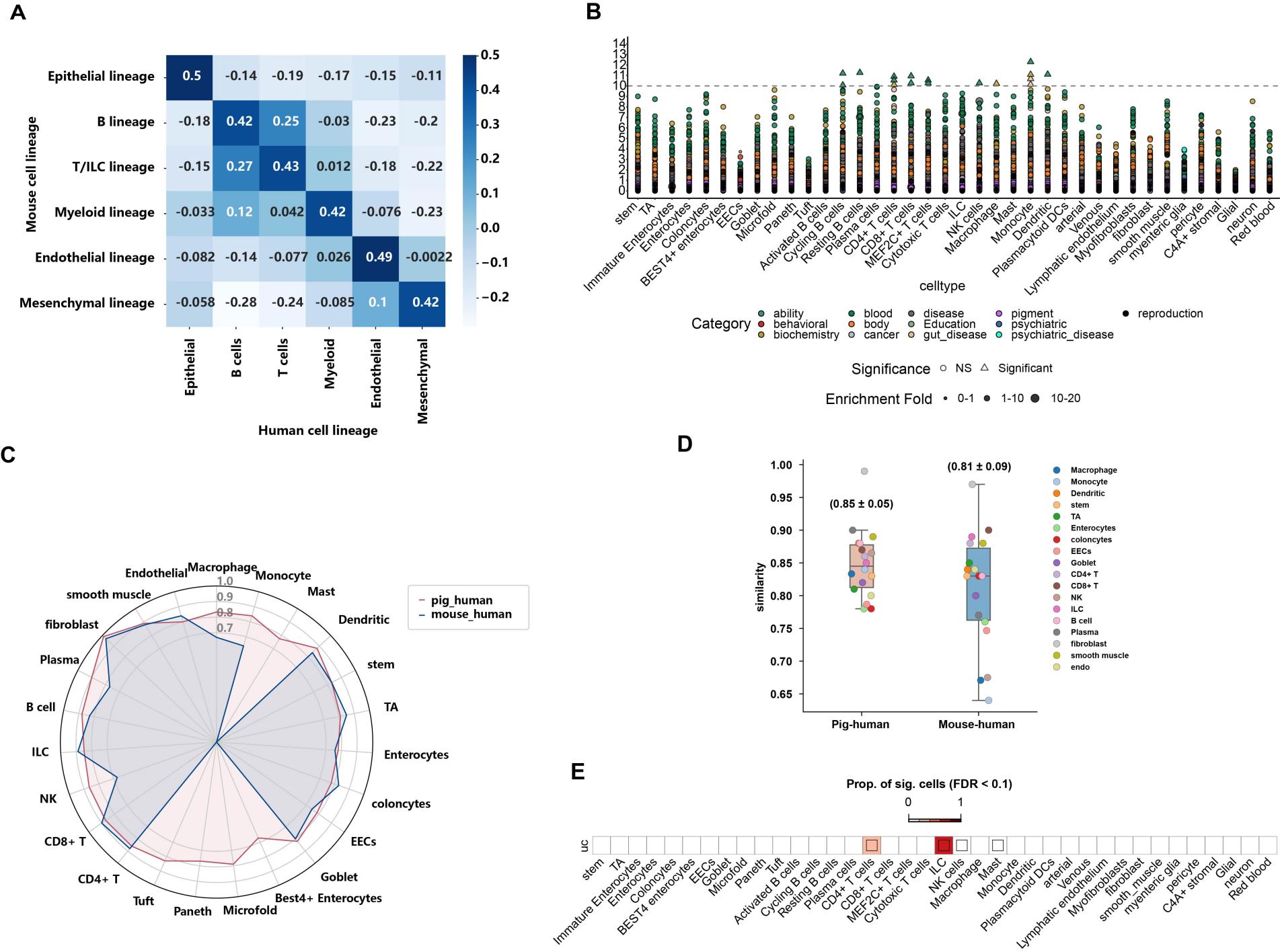


**Figure S5. Correlation of other species and mapping human GWAS summary into IPGCA.**

**A)** Pearson correlation of gene expression between human and mouse cell lineages. **B)** Enrichment scores of LDSC-seg analysis for 176 diverse diseases and traits related to the gut. The color of the points indicates the disease categories. **C)** Pearson's correlation coefficient (r) of gene expression between human-pig and human-mouse cell types (note that some cell types were not found in specific species). **D)** The box plot shows the DEGs correlation between pig verse human and mouse verse human (each dot represents a shared cell type). **E)** Association of ulcerative colitis (UC) from the GWAS catalog with ILC and CD4^+^ T cells, as identified by scDRS.


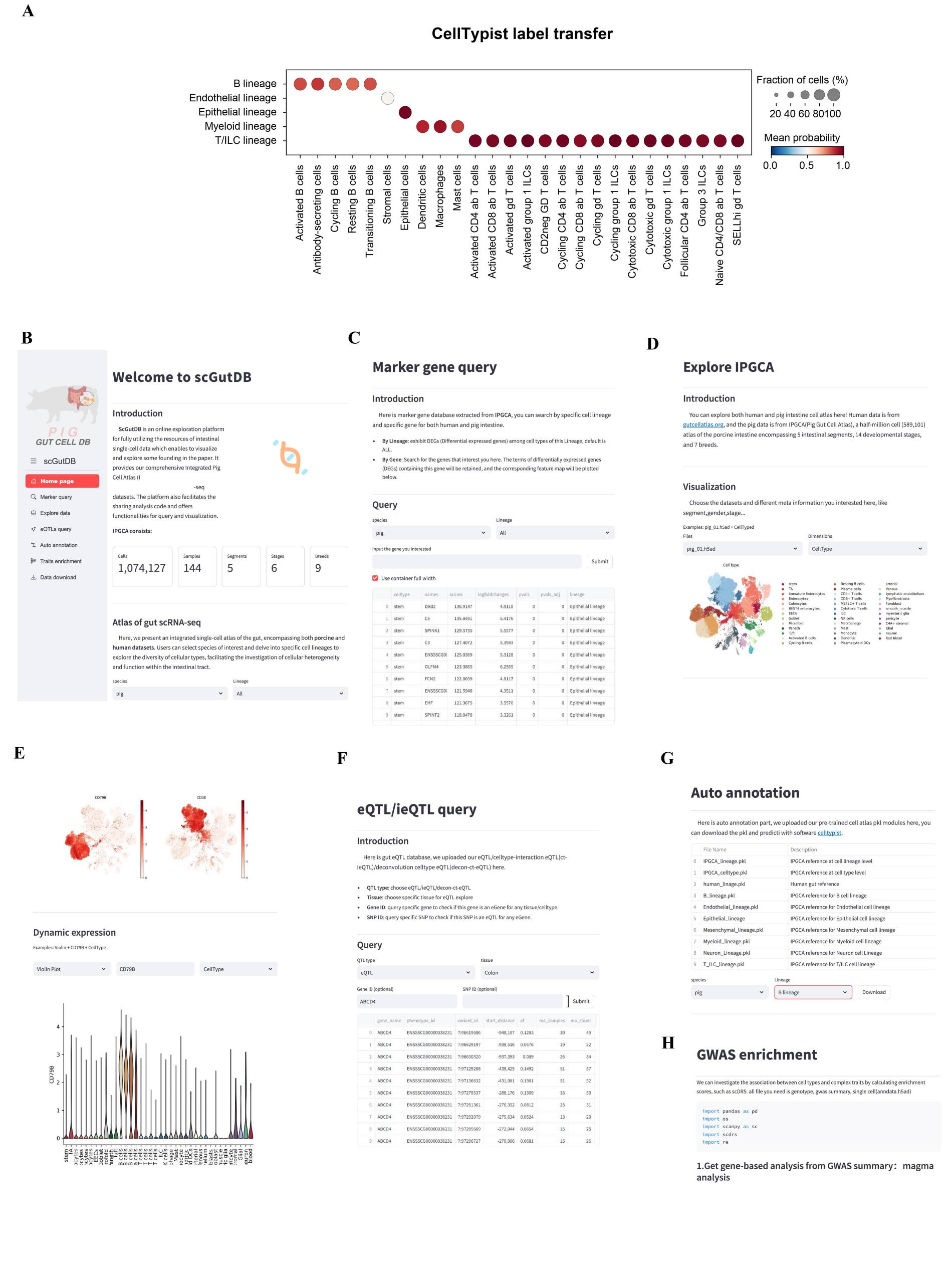


**Figure S6. Information and modules of single cell gut database (scGutDB).**

**A)** The confidence score of celltypist label transfer at the cell lineage level, with the y-axis representing the lineages in the model and the x-axis representing the original annotations. **B)** The home page of scGutDB. **C)** The mark gene query page of scGutDB. **D)** The visualization and exploration page of scGutDB. **E)** The visualization and exploration page of scGutDB, user can plot specific gene here in UMAP or violin plot. **F)** The eQTL query page of scGutDB. **G)** The auto annotation page of scGutDB, user can download the pkl model files here. **H)** The GWAS enrichment page of scGutDB.

**Extend Figure**

**
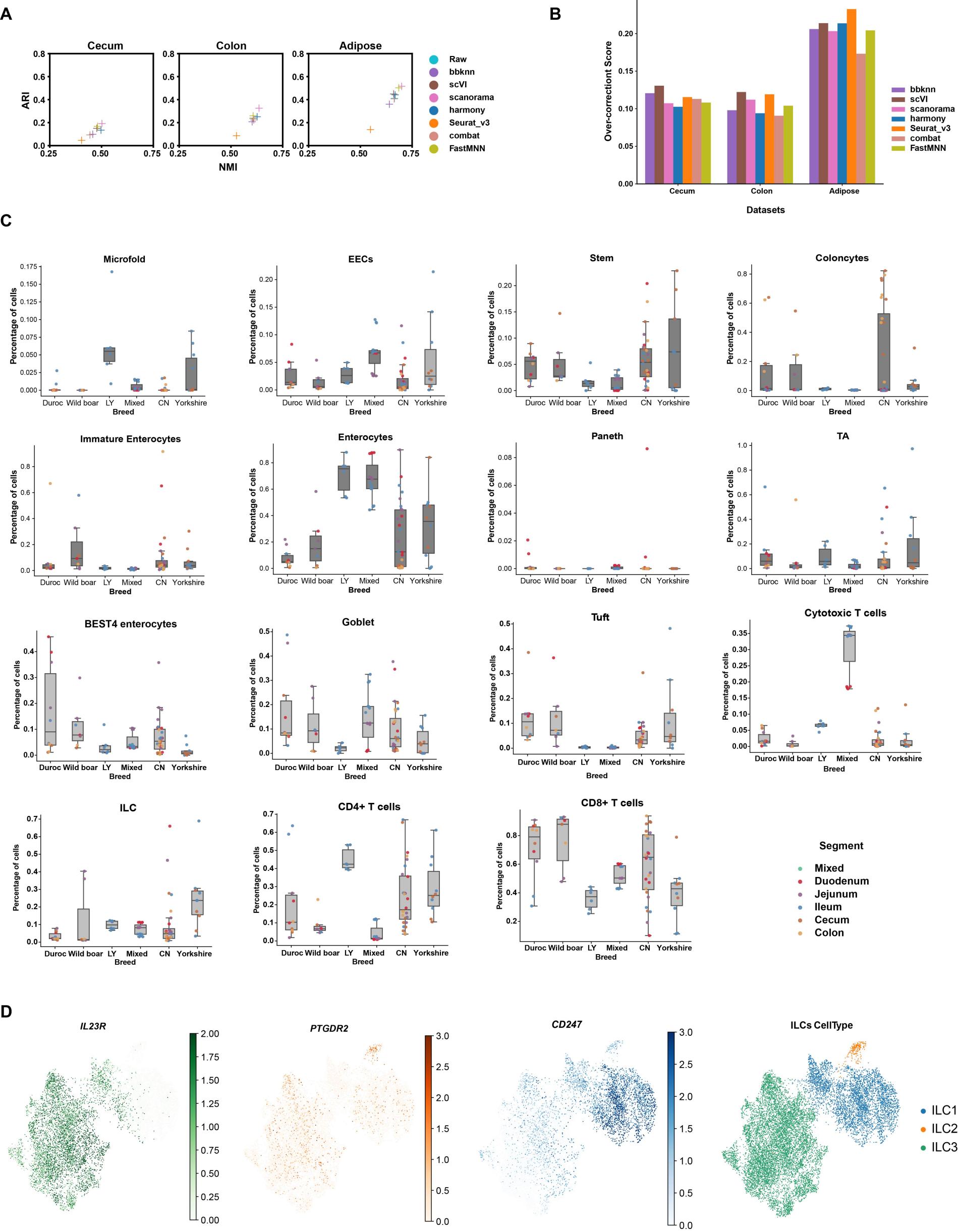
**

**Extend Figure1. Batch effect correction and distribution of cell types.**

**A)** The adjusted rand index (ARI) and normalized mutual information (NMI) calculated for 7 batch correction methods applied to cecum, colon, and adipose tissue datasets. **B)** Over-correction score for 7 batch correction methods applied to cecum, colon, and adipose tissue datasets. **C)** The distributions of epithelial subtypes and immune subtypes along with breed and their distribution pattern of specific segment. **D)** The feature map of the marker genes of ILC1, ILC2, ILC3 and its annotation.


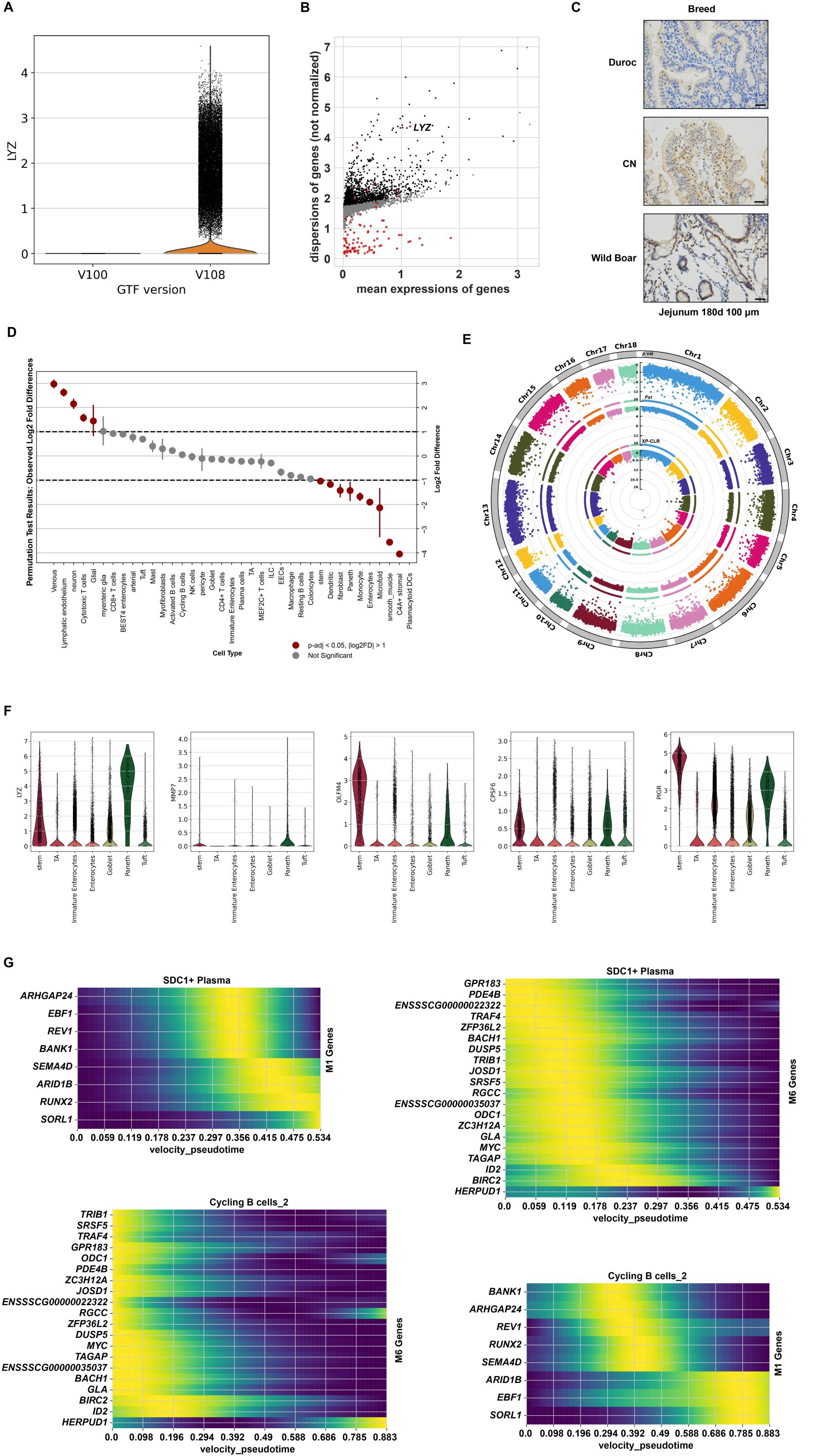


**Extend Figure2. Paneth cell exploration and B lineage activation.**

**A)** The bias of the capture of *LYZ* gene in different gtf versions (v100 and v108). **B)** The difference of genes between the two gtf versions and its distribution in high variable genes of gtf v108 (the red dots represents the different genes). **C)** Immunohistochemistry positive rate across breeds (Duroc, CN and wild boar pigs) sample size is list below. **D)** The cell proportion analysis between CN pigs and Wild boars (1,000 permutations, differences with an absolute log2 fold change greater than 1 were considered significant). **E)** The circular Manhattan plot for selective-sweep signals (outer ring, eigenGWAS -log_10_ (*p*); middle ring, normalized *F*_ST_; inner ring, normalized XP-CLR). **F)** The violin plot of difference epithelial cell types of Paneth cell markers (*LYZ*, *MMP7*) and stemness genes. **G)** The Heatmap of the expression along with velocity pseudotime of core genes from the M1 and M6 module in “*SDC1*_+_ Plasma” and “Cycling B cells_2” trajectory.


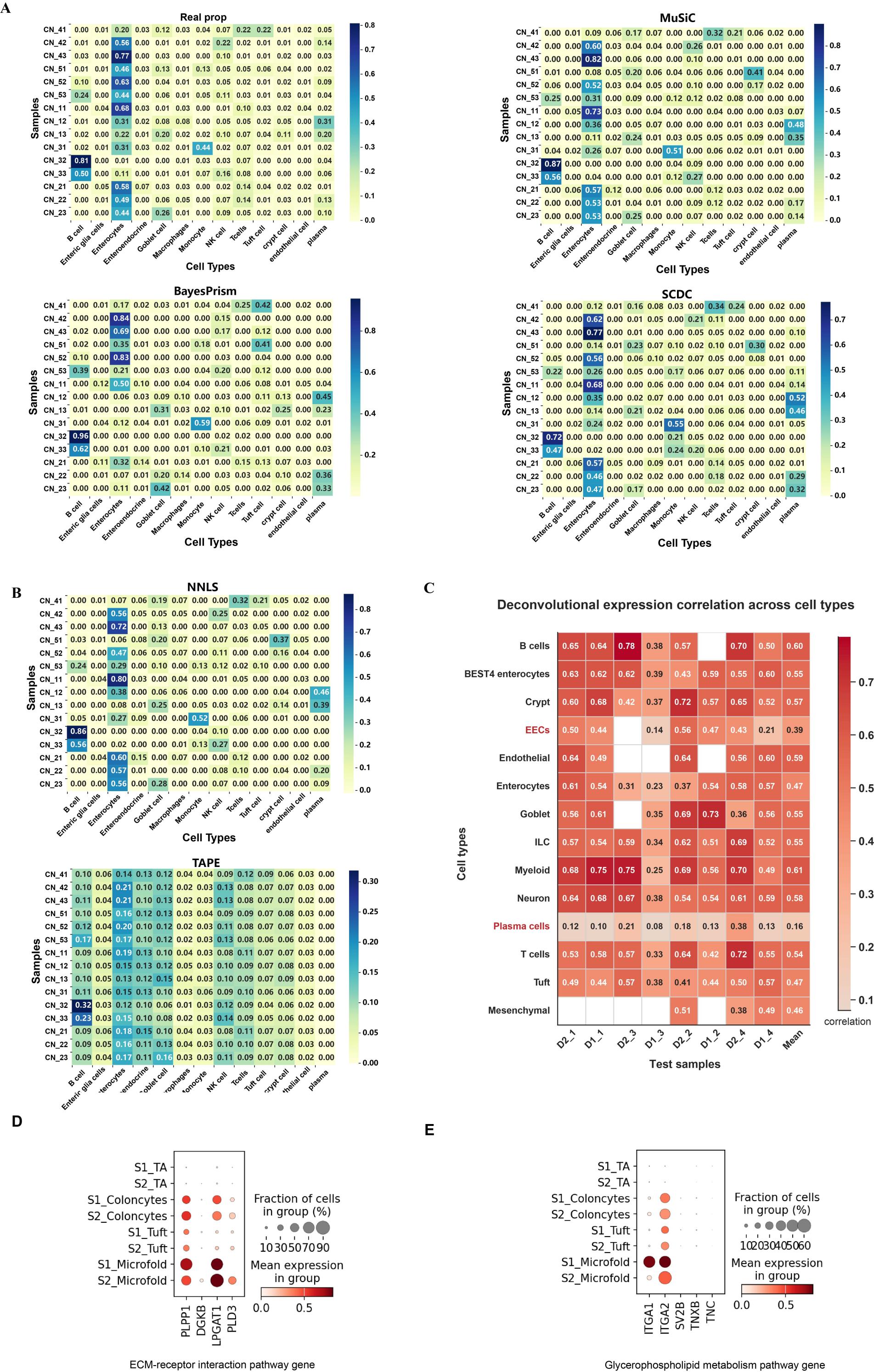


**Extend Figure3. Deconvolution of different method and illustration of pathways enriched by star DEGs between two Duroc lines**

**A**-**B)** The Pearson correlation was calculated to assess the accuracy of five deconvolution methods in estimating cell type proportions compared to the real proportions. **C)** The Pearson’s correlation in estimating cell type expression and some cell types (colored in red) was excluded for subsequent analysis. **D**) The “ECM-receptor interaction pathway (ssa04512)” and **E)** “the glycerophospholipid metabolism pathway (ssa00564)” are two of the most significant pathways identified in the GWAS gene-based analysis enrichment for M_LMA.

**Supplementary Table**

**Table S1** The metadata of collected and generated scRNA-seq datasets.

**Table S2** The marker genes used in the annotation of IPGCA core.

**Table S3** The GO and KEGG term of all breed-specific DEGs.

**Table S4** The sample information extracted from PHARP.

**Table S5** The detailed information of meta-GWAS summary from PigBiobank.

**Table S6** The metadata of collected bulk RNA-seq datasets.

**Table S7** The phenotypic information of two Duroc lines.

**Table S8** The significant co-localization events of complex traits from PigBiobank.

**Table S9** The co-localization events of independent loci between GWAS and three types of eQTLs.

**Table S10** The detailed information of meta-GWAS summary from UK Biobank.
